# Ovarian reserve modulates the impact of vitamin D deficiency on assisted reproductive outcomes in patients undergoing controlled ovarian hyperstimulation

**DOI:** 10.3389/fnut.2024.1486958

**Published:** 2024-12-12

**Authors:** Lina He, Qing Xu, Li Hao, Xu Ran, Yamin Qiu, Jie Lin, Wei Chen

**Affiliations:** ^1^Department of Reproductive Medicine, Zigong Hospital of Women and Children Health Care, Zigong, China; ^2^Department of Urology, Zigong Fourth People’s Hospital, Sichuan, China; ^3^Institute of Precision Medicine, Zigong Academy of Big Data and Artificial Intelligence for Medical Science, Sichuan, China

**Keywords:** vitamin D, controlled ovarian hyperstimulation, *in vitro* fertilization, assisted reproductive, diminished ovarian reserve

## Abstract

**Objective:**

The association between vitamin D deficiency and ovarian reserve-specific outcomes of assisted reproductive technology (ART) remains uncertain. This study aimed to investigate the role of ovarian reserve in the association between basal serum vitamin D levels and ART outcomes in patients undergoing controlled ovarian hyperstimulation (COH).

**Methods:**

A total of 1,333 infertile women undergoing COH cycles were retrospectively analyzed. Patients were divided into a vitamin D deficiency group (serum vitamin D < 20 ng/mL) and a normal vitamin D group. Univariate and multivariate logistic regression analyses were performed to estimate the relationship between vitamin D deficiency and pregnancy outcomes including biochemical pregnancy rate, clinical pregnancy rate, miscarriage rate, and live birth rate in the overall cohort and subgroups with diminished ovarian reserve (DOR) or polycystic ovary syndrome (PCOS).

**Results:**

In the entire participants’ cohort, no correlation between vitamin D deficiency and pregnancy results was observed (*p* > 0.05). However, in the DOR subgroup, vitamin D deficiency was associated with a lower biochemical pregnancy rate (adjusted OR 0.27; 95% CI 0.08–0.90; *p* < 0.01) and clinical pregnancy rate (adjusted OR 0.36; 95% CI 0.14–0.90; *p* < 0.01). No significant differences were observed in pregnancy outcomes among patients with PCOS (*p* > 0.05).

**Conclusion:**

Vitamin D deficiency does not affect pregnancy outcomes in the overall patient population, but it may negatively impact women with DOR potentially leading to poorer pregnancy outcomes. Further studies are needed to clarify the mechanisms involved and the potential use of vitamin D screening and supplementation in specific populations.

## 1 Introduction

Vitamin D is well-known to physicians due to its critical role in calcium and phosphorus metabolism, bone health, and its functions in the immune system (1, 2). Recent molecular studies have revealed that vitamin D exerts its reproductive effects through vitamin D receptors (VDRs) expressed in various reproductive tissues. In the ovary, VDR signaling regulates key steroidogenic enzymes and influences follicular development through both genomic and non-genomic pathways (3). The vitamin D-VDR complex directly modulates gene transcription through vitamin D response elements (VDREs) found in promoter regions of crucial reproductive genes. Calcitriol, which is an active form of vitamin D, may in some positive manner affect the processes of follicle development, endometrial receptivity, and embryonic implantation (4, 5). However, the effects of vitamin D deficiency on assisted reproductive technology (ART), including artificial insemination (AIH) and *in vitro* fertilization-embryo transfer (IVF-ET), and intracytoplasmic sperm injection (ICSI), outcomes remain controversial (5–8). The findings for adequate vitamin D levels and live birth rates were positive in women undergoing IVF in some studies (9, 10), but negative in others (11, 12). There are signs that the consequences of vitamin D deficiency can be additive, under certain conditions, namely, depending on the ovarian reserve (13). Studies have revealed that women with diminished ovarian reserve (DOR) often experience endocrine disturbances in their vitamin D metabolism and signaling, potentially exacerbating their physiological responses to vitamin D deficiency (14). However, in women with polycystic ovary syndrome (PCOS) and relatively higher ovarian reserve, the effect of vitamin D deficiency might be different because of the hormonal and metabolic disorders related to these diseases (15). Recent molecular studies have revealed that vitamin D directly influences reproductive function through several key pathways. Particularly noteworthy is the discovery of specific vitamin D binding sites in the promoter region of the AMH gene, suggesting direct transcriptional regulation of this key marker of ovarian reserve (16). Additionally, vitamin D has been shown to modulate endometrial immune responses critical for implantation, including regulation of decidualization-related genes and local cytokine production (17). In granulosa cells, vitamin D influences steroidogenesis through both direct transcriptional regulation of steroidogenic enzymes and rapid non-genomic signaling pathways (18).

The impact of vitamin D deficiency may vary depending on ovarian reserve status (13). Women with DOR often exhibit altered vitamin D metabolism and signaling pathways, potentially influencing their response to vitamin D deficiency (14). Conversely, in women with PCOS or relatively high ovarian reserve (OR), the impact of vitamin D deficiency may differ due to the distinct endocrine and metabolic profiles associated with these conditions (15). Recent reports have suggested a possible relationship between vitamin D and ovarian reserve profile, highlighting its importance in folliculogenesis (19). Specific binding sites of vitamin D have been strictly identified in the promoter of the human AMH gene, which suggests that vitamin D has a direct influence on AMH gene expression (20). Nevertheless, up to now, whether the deficiency of vitamin D could influence pregnancy outcomes, particularly for the OR-specific population, remains controversial. This study aimed to provide additional evidence by exploring the association of vitamin D deficiency with pregnancy outcomes in patients with controlled ovarian hyperstimulation (COH) cycles.

## 2 Materials and methods

### 2.1 Participants

This retrospective study examined data from female patients who received COH cycles at Zigong Hospital of Women and Children Health Care between November 2019 and May 2024. The study protocol was approved by the Institutional Ethics Committee of this hospital (approval number: 2024IECA04). The inclusion criteria are: (1) women undergoing COH cycles for ART, including AIH, IVF-ET, and ICSI; (2) patients with available baseline serum 25-hydroxyvitamin D [25(OH)D] levels; (3) patients with complete data on biochemical pregnancy outcomes; and (4) patients with follow-up data for a clinical pregnancy, miscarriage, or live birth outcomes. Those patients were excluded: (1) patients whose follow-up data for any specific outcome (clinical pregnancy, miscarriage, and live birth) were incomplete or the follow-up period for these outcomes had not ended were excluded for the corresponding outcome; (2) patients with known endocrine disorders other than PCOS (e.g., hyperprolactinemia and thyroid disorders) that could confound the study results; (3) patients with severe systemic diseases or conditions (e.g., cancer, uncontrolled diabetes, and severe cardiovascular diseases) that might independently affect pregnancy outcomes; (4) patients on medications known to interfere with vitamin D metabolism (e.g., anticonvulsants and glucocorticoids) within 3 months prior to the study; (5) patients with prior history of recurrent pregnancy loss (defined as three or more consecutive miscarriages), which might affect the study outcomes independently; and (6) patients undergoing donor egg cycles, as these cycles have different dynamics compared to autologous cycles. All patients provided written informed consent for the use of their clinical data for research purposes at the time of receiving medical treatment.

### 2.2 Baseline assessments

The baseline serum levels of serum 25(OH)D, follicle-stimulating hormone (FSH), estradiol, progesterone, prolactin, luteinizing hormone (LH), testosterone, anti-Müllerian hormone (AMH), thyroid-stimulating hormone (TSH), cancer antigen 125 (CA125), fasting plasma glucose, and fasting insulin were measured within 4 weeks before the initiation of the COH cycle. The serum 25(OH)D levels were determined using a chemiluminescence immunoassay method with the iFlash 3,000 analyzer (Shenzhen YHLO Biotech Co., Ltd., China).

### 2.3 Group classification

Participants were categorized into two groups based on their serum vitamin D levels: the vitamin D deficiency group (<20 ng/mL) and the normal group (≥20 ng/mL) (21). Diminished ovarian reserve (DOR) was defined according to one of the following criteria: (i) basal FSH level ≥ 10 mIU/ml or, (ii) basal AMH ≤1.1 ng/mL or, (iii) AFC ≤ 5 (22). Polycystic ovary syndrome (PCOS) was diagnosed based on the Rotterdam criteria (23).

### 2.4 Outcome measures

The primary outcomes of interest were biochemical pregnancy rate, clinical pregnancy rate, miscarriage rate, and live birth rate. Biochemical pregnancy was defined as a positive serum *β*-human chorionic gonadotropin (β-hCG) level ≥ 5 mIU/mL after 14 days of operation. Clinical pregnancy was confirmed by the presence of a gestational sac with fetal heartbeat on ultrasound examination above 6 weeks of gestation. Miscarriage was defined as pregnancy loss before 28 weeks of gestation. Live birth rate refers to the delivery of a live infant after 28 weeks of gestation.

### 2.5 Statistical analysis

Continuous data were expressed as median, 25th, and 75th percentiles, and differences between groups were subjected to the Wilcoxon rank-sum test. Categorical data were expressed as frequencies and percentages, and differences in size between groups were assessed using chi-square analysis or, if the expected range was less than 5, by Fisher’s exact test. The comparison of the characteristics in the overall cohort and the subgroups based on DOR and PCOS was conducted by performing univariable binary logistic regression analysis where potential risk factors were examined in relation to pregnancy outcomes using odds ratio (OR) and 95% confidence interval (CI). To control for potential confounders, a multivariable analysis of the relationship between vitamin D and pregnancy outcomes in the pregnant women in the overall cohort and the subgroups was performed using data distinguished in the univariable analysis. Statistical significance was set at a two-sided *p*-value of <0.05. The analysis was conducted using R software (version 4.3.0, R Core Team, R Foundation for Statistical Computing).

## 3 Results

### 3.1 Patient characteristics

A total of 2,699 patients were retrieved from the institutional database. After excluding the patients with missing data on 25(OH)D levels and clinical outcomes, the baseline characteristics of the 1,333 remaining patients, stratified by 25(OH)D levels are shown in Table 1. All patients in the study were Chinese. The deficiency group (*n* = 937, 70.3%) had significantly lower levels of education (50.60% vs. 56.25% above bachelor’s degree, *p* = 0.01), lower prevalence of PCOS (6.40% vs. 12.88%, *p* < 0.01), and lower serum levels of estradiol (42.10pg/ml vs. 45.35pg/ml, *p* = 0.01) but higher serum level of FSH (7.20mIU/ml vs. 6.95mIU/ml, *p* = 0.02) than the normal group (*n* = 396, 29.7%). While there was no significant statistical difference in the gender of newborns between the two groups (*p* = 0.75).

**Table 1:** Basic characteristics between patients with 25(OH)D deficiency and normal level.

Characteristics		Normal (*n* = 396)	Deficiency (*n* = 937)	*p*-value
Age, years old		32 (29–35)	32 (29–35)	0.98
BMI, kg/m^2^		21.60 (19.80–24.08)	21.40 (19.80–23.70)	0.27
Education	PS	11 (2.86)	40 (4.34)	0.01
	Junior MS	123 (32.03)	291 (31.60)	
	Senior MS	34 (8.85)	124 (13.46)	
	Bachelor	207 (53.91)	460 (49.95)	
	Master/ Doctor	9 (2.34)	6 (0.65)	
Progression, years		3 (2–5)	3 (2–5)	0.21
Infertility type	Primary	190 (47.98)	460 (49.09)	0.71
	Secondary	206 (52.02)	477 (50.91)	
PCOS		51 (12.88)	60 (6.40)	0.00
DOR		61 (15.64)	122 (13.59)	0.33
COH cycle	AIH	9 (2.27)	42 (4.48)	0.20
	IVF-ET/ICSI Frozen	184 (46.46)	421 (44.93)	
	IVF-ET/ICSI Fresh	203 (51.26)	474 (50.59)	
COH protocol	Antagonist	144 (38.2)	331 (36.86)	0.70
	Agonist	53 (14.06)	116 (12.92)	
	Other	180 (47.75)	451 (50.22)	
FSH, mIU/ml		6.95 (5.80–8.30)	7.20 (6.30–8.60)	0.02
Estradiol, pg./ml		45.35 (34.70–57.08)	42.10 (32.60–53.60)	0.01
Progestogen, ng/ml		0.46 (0.36–0.65)	0.48 (0.33–0.63)	0.17
Prolactin, ng/ml		17.75 (13.04–23.8)	18.8 (14.21–25.08)	0.07
LH, mIU/ml		4.20 (2.90–60)	3.80 (2.70–5.10)	0.01
Testosterone, ng/ml		0.27 (0.17–0.39)	0.25 (0.17–0.35)	0.07
AMH, ng/ml		3.31 (2.07–5.94)	3.35 (1.96–5.12)	0.99
TSH, mIU/L		2.62 (1.78–3.69)	2.41 (1.74–3.38)	0.64
CA125, U/mL		18.30 (12.50–29.70)	18.15 (12.90–30.60)	0.70
FPG, mmol/L		5.19 (4.95–5.51)	5.21 (4.96–5.51)	0.60
FINS, uIU/ml		8.64 (5.50–13.03)	9.59 (5.99–13.31)	0.25
AFC		16 (10–24)	15 (10–24)	0.28
25(OH)D, ng/ml		23.5 (21.20–26.30)	15.1 (12.15–17.50)	<0.01
Biochemical pregnancy		129 (63.86)	311 (62.58)	0.75
Clinical pregnancy		94 (48.21)	226 (48.71)	0.95
Miscarriage		19 (20.21)	42 (18.75)	0.12
Live birth		34 (36.17)	108 (48.21)	0.12
	Male	19 (55.88)	57 (52.78)	0.75
	Female	15 (44.12)	51 (47.22)	

PS, primary school; MS, middle school; BMI, body mass index; PCOS, polycystic ovary syndrome; DOR, diminished ovarian reserve; AIH, artificial insemination; IVF-ET, in vitro fertilization-embryo transfer; COH, controlled ovarian hyperstimulation; AMH, anti-Mullerian hormone; FSH, follicle-stimulating hormone; LH, luteinizing hormone; TSH, thyroid-stimulating hormone; FPG, fasting plasma glucose; FINS, fasting insulin; AFC, antral follicle count.

### 3.2 Pregnancy outcomes in the overall cohort

**Figure 1:**
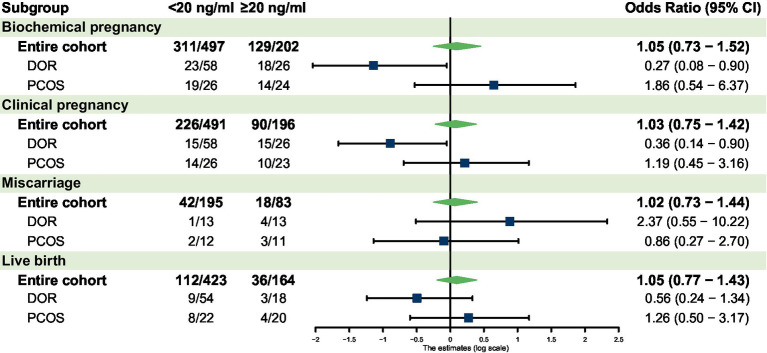
Forest plot for the subgroup analyses. AMH, anti-Müllerian hormone; DOR, diminished ovarian reserve; PCOS, polycystic ovary syndrome; CI, confidence interval.

In the overall cohort, vitamin D deficiency was not significantly associated with biochemical pregnancy rate, clinical pregnancy rate, miscarriage rate, or live birth rate compared to normal vitamin D levels (Table 2). The results of the univariable analysis for each potential confounder are listed in Supplementary Tables 1–4. Multivariable logistic regression analysis confirmed no significant association between vitamin D status and biochemical pregnancy, clinical pregnancy, miscarriage, or live birth in the overall cohort (Figure 1).

**Table 2:** Pregnancy, miscarriage, and live birth rates between patients with 25(OH)D deficiency and normal level.

Indicator	Biochemical pregnancy		Clinical pregnancy		Miscarriage		Live birth	
	Event/total (%)	*p*	Event/total (%)	*p*	event/total (%)	*p*	event/total (%)	*p*
Entire cohort								
Normal	129/202 (63.86%)	0.82	90/196 (45.92%)	0.98	18/83 (21.69%)	0.97	36/164 (21.95%)	0.26
Deficiency	311/497 (62.58%)		226/491 (46.03%)		42/195 (21.54%)		112/423 (26.48%)	
Patients with PCOS								
Normal	14/24 (58.33%)	0.42	10/23 (43.48%)	0.47	3/11 (27.27%)	0.59	4/20 (20%)	0.20
Deficiency	19/26 (73.08%)		14/26 (53.85%)		2/12 (16.67%)		8/22 (36.36%)	
Patient with DOR								
Normal	18/26 (69.23%)	0.02	15/26 (57.69%)	0.01	4/13 (30.77%)	0.16	3/18 (16.67%)	0.63
Deficiency	23/58 (39.66%)		15/58 (25.86%)		1/13 (7.69%)		9/54 (16.67%)	

DOR, diminished ovarian reserve; PCOS, polycystic ovary syndrome.

### 3.3 Pregnancy outcomes in the ovarian reserve-specific group

Table 3 shows the univariate regression for patients with 25(OH)D deficiency compared to the normal level. For patients with DOR, vitamin D deficiency was associated with significantly lower biochemical pregnancy rates (*p* = 0.02 in Table 2) and clinical pregnancy rates (*p* = 0.01 in Table 2). Multivariable analysis confirmed lower odds of biochemical pregnancy (OR 0.27, 95% CI 0.08–0.90, *p* = 0.03) and clinical pregnancy (OR 0.36, 95% CI 0.14–0.9, *p* = 0.02) in the deficient group (Figure 1). No significant differences were observed for miscarriage or live birth.

**Table 3:** Univariable regression results for patients with 25(OH)D deficiency compared to normal level.

Predictor	Beta	SE	Wald value	OR (95% CI)	p-value
Clinical pregnancy					
Entire cohort	0.03	0.17	0.18	1.03 (0.74–1.44)	0.86
DOR	−1.36	0.50	−2.74	0.26 (0.10–0.68)	0.01
PCOS	0.24	0.57	0.42	1.27 (0.41–3.92)	0.67
Biochemical pregnancy					
Entire cohort	−0.06	0.17	−0.32	0.95 (0.67–1.33)	0.75
DOR	−1.23	0.50	−2.45	0.29 (0.11–0.78)	0.01
PCOS	0.66	0.61	1.09	1.94 (0.59–6.36)	0.27
Live birth					
Entire cohort	0.09	0.31	0.30	1.10 (0.60–2.01)	0.76
DOR	1.63	1.19	1.37	5.09 (0.5–52.29)	0.17
PCOS	0.81	1.02	0.79	2.25 (0.3–16.63)	0.43
Miscarriage					
Entire cohort	−0.50	0.25	−1.96	0.61 (0.37–1.00)	0.05
DOR	−2.01	0.92	−2.18	0.13 (0.02–0.82)	0.03
PCOS	−0.85	0.83	−1.02	0.43 (0.08–2.17)	0.31

The normal group (≥20 ng/mL) was set as the controlled group. S.E., standard error; OR, odds ratio; CI, confidence interval.

In patients with PCOS, no statistically significant differences were observed in ART outcomes between vitamin D deficient and normal groups in both univariable and multivariable analyses. However, the small sample size limits the interpretation of these results. The results of the multivariable analysis for each outcome are provided in Supplementary Tables 5–8.

## 4 Discussion

Our study reveals a relationship between vitamin D status and ART outcomes that varies significantly based on ovarian reserve profiles. This heterogeneity in response suggests that the role of vitamin D in reproductive success is more nuanced than previously understood and may require personalized approaches to supplementation based on individual patient characteristics. In the overall cohort, vitamin D status demonstrated no relationship with ART efficacy regarding biochemical pregnancy, clinical pregnancy, miscarriage, and live birth rates. This result is consistent with some previous research suggesting that vitamin D levels do not influence IVF outcomes in general (5, 10–12, 14). A prospective study by Cai et al. (24) reported that the levels of both free and total 25(OH)D measured at the time of embryo transfer showed no association with successful implantation in women undergoing fresh transfers following ovarian hyperstimulation. Van de Vijver et al. (12) also suggested that the routine measurement of vitamin D levels in patients undergoing frozen–thawed cycles may not be warranted. However, our results contrast with other reports indicating a positive association between vitamin D sufficiency and IVF success (12, 20). A case–control study by Woo et al. reported that vitamin D supplementation may significantly prevent preterm birth in black women who are at high risk for vitamin D deficiency (5).

When considering different ovarian reserve statuses, patients with DOR exhibited significantly lower rates of both biochemical and clinical pregnancy when vitamin D was deficient. This finding contrasts with the aforementioned studies and aligns more closely with the hypothesized positive impacts of vitamin D on reproductive health. The negative effect of vitamin D deficiency in DOR patients might be explained by the potential role of vitamin D in ovarian steroidogenesis and follicular development (25). DOR patients have a depleted follicular pool and may experience poor oocyte quality (26). It has been suggested that vitamin D deficiency may exacerbate these conditions, leading to poorer ART outcomes. Moreover, vitamin D has been associated with endometrial receptivity, which may explain the lower pregnancy rates observed in DOR patients with vitamin D deficiency (27).

However, we did not find a relationship between vitamin D status and live birth rates in the DOR group, despite the substantial differences in pregnancy rates between the two groups. This indicates that other factors may influence the transition from pregnancy to live birth in these patients, suggesting the need for further studies with adequate sample sizes to capture differences in live birth rates.

Our study did not establish a significant effect of vitamin D status on ART outcomes in women with PCOS. However, the small sample size in this subgroup limits the interpretability of these results. Previous investigations have indicated that patients with PCOS may have a higher likelihood of experiencing vitamin D deficiency and that this deficiency could potentially contribute to insulin resistance and other metabolic derangements associated with PCOS (9). Research involving larger sample sizes in PCOS groups is necessary to clarify the impact of vitamin D on ART effectiveness.

The observed heterogeneity in vitamin D responses across patient subgroups suggests that the influence of vitamin D on reproductive outcomes may be moderated by ovarian reserve and hormonal balance (28). The vitamin D endocrine system plays a role in several reproductive processes through multiple molecular pathways. In ovarian tissue, vitamin D receptors regulate steroidogenic enzyme expression and AMH production through direct transcriptional control (29). Studies have identified specific vitamin D response elements in the promoter regions region of genes crucial for folliculogenesis (30, 31). In the endometrium, vitamin D modulates local immune responses and inflammatory markers essential for implantation, including the regulation of decidualization-related genes and cytokine production (32). This includes roles in steroidogenesis, folliculogenesis, embryo implantation, and placental function (25, 33). The data from patients with DOR suggest that vitamin D may be beneficial for achieving optimal outcomes in the number of available follicles due to its impact on AMH, follicular fluid, or oocyte quality (34). Conversely, in patients with PCOS and high AMH levels, the effect of vitamin D on ovarian function appears to be more complex, and the feedback mechanisms regulating this interaction remain poorly understood (35, 36). The observed downward trends in miscarriage rates associated with vitamin D deficiency in the univariable analysis, which were not evident in the multivariable analysis, raise questions about the role of vitamin D in early pregnancy maintenance. However, these findings should be interpreted with caution, emphasizing the need for further studies to establish the variable impacts of vitamin D at different reproductive phases.

Several limitations of the current study should be noted. First, due to its retrospective nature, this investigation cannot establish causal relationships between vitamin D deficiency and pregnancy outcomes. Despite the large overall sample size, the small numbers in the DOR and PCOS subgroups may result in false positive or false negative findings due to insufficient statistical power. Second, the present research only assessed vitamin D status cross-sectionally before the ART cycle was initiated. Fluctuations in vitamin D levels throughout the cycle and during pregnancy were not examined, even though significant changes occurred during these periods. Third, we lacked information regarding the duration of vitamin D deficiency or the impact of vitamin D supplementation, which may have affected the observed relationships. Additionally, some patients were still under follow-up at the time of analysis, and clinical outcomes were limited to those patients who had completed their follow-up successfully. Furthermore, although baseline cycle and COH protocol categories did not show significant differences between groups, variability in treatment protocols may have introduced biases and potential confounding effects. Finally, other potential confounding factors—such as dietary habits, sun exposure, genetic factors, and comorbidities—were not considered and may interact with vitamin D deficiency to influence outcomes (37). While these limitations are important to acknowledge, they should be viewed in the context of our study’s significant contributions to understanding vitamin D’s role in reproduction.

Our findings underscore the importance of personalized medicine approaches in fertility treatment, advocating for interventions tailored to specific patient characteristics, such as ovarian reserve status, when evaluating the need for vitamin D assessment and supplementation in ART protocols. For women with DOR, vitamin D screening and potential supplementation may be particularly crucial given the observed negative impact of deficiency on pregnancy rates in this group. However, the lack of significant effects in other patient populations suggests that universal vitamin D supplementation may not be warranted. Based on these findings, prospective cohort studies with larger sample sizes, especially within the subgroups of interest, and thorough evaluations of confounding factors and effect measures are recommended to further affirm and generalize these results. Such studies should also investigate the timing and dosage of effective vitamin D supplementation, as well as potential biomarkers for the observed effects. Additionally, further investigations into the correlation between vitamin D and other indicators of ovarian reserve and activity, including follicular fluid and embryo parameters, could provide valuable insights.

## 5 Conclusion

The results of the present study did not indicate that vitamin D deficiency is associated with poor pregnancy outcomes during the COH cycle in the entire population, while it could be disadvantageous in patients with DOR. Our results suggest the importance of considering individual patient characteristics when evaluating the potential impact of vitamin D on reproductive outcomes.

## Data Availability

The data analyzed in this study is subject to the following licenses/restrictions: the datasets used and/or analyzed during the current study are available from the corresponding author on reasonable request. Requests to access these datasets should be directed to Lina He, linahe.repro@outlook.com; Wei Chen, cweimed@gmail.com.
