# Supplementary material for: Ovarian reserve modulates the impact of vitamin D deficiency on assisted reproductive outcomes in patients undergoing controlled ovarian hyperstimulation
Source: Front Nutr. 2024 Dec 12;11:1486958. doi: 10.3389/fnut.2024.1486958 (PMC11670746; doi:10.3389/fnut.2024.1486958)
Supplement: Supplementary file 1 [file Table_1.DOCX]

**Supplementary files**

**Contents**

[sTable 1. Univariable regression results of clinical pregnancy 2](#_Toc169777159)

[sTable 2. Univariable regression results of biochemical pregnancy 7](#_Toc169777160)

[sTable 3. Univariable regression results of miscarriage 12](#_Toc169777161)

[sTable 4. Univariable regression results of live birth 17](#_Toc169777162)

[sTable 5. Multivariable regressions for clinical pregnancy 22](#_Toc169777163)

[sTable 6. Multivariable regressions for biochemical pregnancy 23](#_Toc169777164)

[sTable 7. Multivariable regressions for miscarriage 24](#_Toc169777165)

[sTable 8. Multivariable regressions for live birth 25](#_Toc169777166)

**Abbreviations:** S.E., standard error; OR, odds ratio; CI, confidence interval; PS, primary school; MS, middle school; BMI, KG/M2, kg/m^2^, body mass index; PCOS, polycystic ovary syndrome; DOR, diminished ovarian reserve; COH, controlled ovarian hyperstimulation; FSH, follicle-stimulating hormone; LH, luteinizing hormone; TSH, thyroid stimulating hormone; FPG, fasting plasma glucose; FINS, fasting insulin; AFC, antral Follicle Count

# sTable 1. Univariable regression results of clinical pregnancy

| **Predictor** | **Beta** | **SE** | **Wald** | **OR** | **Lower CI** | **Upper CI** | **P** |
| --- | --- | --- | --- | --- | --- | --- | --- |
| **Entire cohort** |  |  |  |  |  |  |  |
| (Intercept) | 1.28 | 0.42 | 3.02 | 3.60 | 1.57 | 8.27 | 0.00 |
| Age, years old | -0.01 | 0.01 | -0.78 | 0.99 | 0.96 | 1.02 | 0.43 |
| (Intercept) | 0.97 | 0.31 | 3.10 | 2.64 | 1.43 | 4.89 | 0.00 |
| Education Junior MS | 0.16 | 0.33 | 0.48 | 1.17 | 0.61 | 2.26 | 0.63 |
| Education Senior MS | -0.40 | 0.35 | -1.13 | 0.67 | 0.33 | 1.34 | 0.26 |
| Education Bachelor | -0.02 | 0.33 | -0.07 | 0.98 | 0.52 | 1.85 | 0.95 |
| Education MD/PhD | 0.04 | 0.66 | 0.06 | 1.04 | 0.28 | 3.81 | 0.95 |
| (Intercept) | 0.98 | 0.09 | 11.09 | 2.65 | 2.23 | 3.15 | 0.00 |
| Infertility type (secondary) | -0.04 | 0.12 | -0.36 | 0.96 | 0.75 | 1.22 | 0.72 |
| (Intercept) | 0.93 | 0.10 | 9.38 | 2.54 | 2.09 | 3.09 | 0.00 |
| Progression, years | 0.00 | 0.02 | 0.17 | 1.00 | 0.96 | 1.04 | 0.87 |
| (Intercept) | 0.97 | 0.45 | 2.16 | 2.64 | 1.09 | 6.37 | 0.03 |
| BMI, KG/M2 | 0.00 | 0.02 | -0.04 | 1.00 | 0.96 | 1.04 | 0.97 |
| (Intercept) | 0.91 | 0.17 | 5.32 | 2.49 | 1.78 | 3.49 | 0.00 |
| FSH, mIU/ml | 0.01 | 0.02 | 0.43 | 1.01 | 0.97 | 1.05 | 0.67 |
| (Intercept) | 0.89 | 0.11 | 7.80 | 2.42 | 1.94 | 3.03 | 0.00 |
| Estradiol, pg/ml | 0.00 | 0.00 | 1.01 | 1.00 | 1.00 | 1.01 | 0.31 |
| (Intercept) | 0.98 | 0.07 | 14.66 | 2.65 | 2.33 | 3.02 | 0.00 |
| Progestogen, ng/ml | 0.01 | 0.03 | 0.56 | 1.01 | 0.97 | 1.07 | 0.57 |
| (Intercept) | 0.90 | 0.13 | 7.16 | 2.47 | 1.93 | 3.16 | 0.00 |
| Prolactin, ng/ml | 0.00 | 0.01 | 0.91 | 1.00 | 0.99 | 1.01 | 0.36 |
| (Intercept) | 0.86 | 0.11 | 7.91 | 2.37 | 1.91 | 2.94 | 0.00 |
| LH.base | 0.03 | 0.02 | 1.30 | 1.03 | 0.99 | 1.07 | 0.19 |
| (Intercept) | 1.01 | 0.07 | 15.36 | 2.75 | 2.42 | 3.13 | 0.00 |
| Testosterone, ng/ml | 0.00 | 0.02 | 0.27 | 1.00 | 0.97 | 1.04 | 0.79 |
| (Intercept) | 0.79 | 0.10 | 7.86 | 2.20 | 1.81 | 2.68 | 0.00 |
| AMH.base | 0.04 | 0.02 | 1.98 | 1.04 | 1.00 | 1.08 | 0.05 |
| (Intercept) | 1.03 | 0.10 | 10.15 | 2.79 | 2.29 | 3.41 | 0.00 |
| TSH, mIU/L | -0.03 | 0.03 | -0.98 | 0.97 | 0.92 | 1.03 | 0.33 |
| (Intercept) | 0.97 | 0.08 | 11.67 | 2.64 | 2.24 | 3.11 | 0.00 |
| CA125, U/ML | 0.00 | 0.00 | -0.36 | 1.00 | 1.00 | 1.00 | 0.72 |
| (Intercept) | 1.33 | 0.37 | 3.60 | 3.78 | 1.83 | 7.81 | 0.00 |
| FPG, mmol/L | -0.07 | 0.07 | -1.05 | 0.93 | 0.81 | 1.06 | 0.29 |
| (Intercept) | 0.94 | 0.15 | 6.17 | 2.57 | 1.90 | 3.46 | 0.00 |
| FINS, uIU/ml | 0.01 | 0.01 | 0.62 | 1.01 | 0.98 | 1.03 | 0.54 |
| (Intercept) | 0.78 | 0.11 | 6.97 | 2.19 | 1.76 | 2.73 | 0.00 |
| AFC | 0.01 | 0.01 | 1.90 | 1.01 | 1.00 | 1.02 | 0.06 |
| (Intercept) | 1.01 | 0.11 | 8.87 | 2.74 | 2.19 | 3.42 | 0.00 |
| 25(OH)D≥ 20 ng/ml | -0.08 | 0.13 | -0.56 | 0.93 | 0.71 | 1.21 | 0.57 |
| (Intercept) | -1.41 | 0.35 | -4.00 | 0.24 | 0.12 | 0.49 | 0.00 |
| COH cycle Frozen | 1.51 | 0.36 | 4.18 | 4.54 | 2.24 | 9.24 | 0.00 |
| COH cycle Fresh | 4.10 | 0.39 | 10.62 | 60.45 | 28.35 | 128.89 | 0.00 |
| Cyle.type4 | 1.41 | 1.46 | 0.97 | 4.10 | 0.24 | 71.36 | 0.33 |
| (Intercept) | 2.56 | 0.18 | 14.40 | 12.97 | 9.15 | 18.39 | 0.00 |
| Protocol type Agonist | -1.11 | 0.27 | -4.18 | 0.33 | 0.20 | 0.55 | 0.00 |
| Protocol type Other | -2.49 | 0.20 | -12.77 | 0.08 | 0.06 | 0.12 | 0.00 |
| **Patient with DOR** |  |  |  |  |  |  |  |
| (Intercept) | 2.01 | 1.09 | 1.84 | 7.44 | 0.88 | 63.15 | 0.07 |
| Age, years old | -0.03 | 0.03 | -0.92 | 0.97 | 0.92 | 1.03 | 0.36 |
| (Intercept) | 2.08 | 1.06 | 1.96 | 8.00 | 1.00 | 63.96 | 0.05 |
| Education Junior MS | -1.05 | 1.09 | -0.96 | 0.35 | 0.04 | 2.98 | 0.34 |
| Education Senior MS | -1.77 | 1.13 | -1.56 | 0.17 | 0.02 | 1.57 | 0.12 |
| Education Bachelor | -0.96 | 1.10 | -0.88 | 0.38 | 0.04 | 3.28 | 0.38 |
| Education MD/PhD | -1.39 | 1.62 | -0.86 | 0.25 | 0.01 | 5.98 | 0.39 |
| (Intercept) | 1.08 | 0.27 | 4.07 | 2.95 | 1.75 | 4.96 | 0.00 |
| Infertility type (secondary) | -0.09 | 0.33 | -0.28 | 0.91 | 0.48 | 1.74 | 0.78 |
| (Intercept) | 1.19 | 0.25 | 4.83 | 3.30 | 2.03 | 5.36 | 0.00 |
| Progression, years | -0.04 | 0.04 | -0.97 | 0.96 | 0.88 | 1.04 | 0.33 |
| (Intercept) | -0.10 | 1.42 | -0.07 | 0.91 | 0.06 | 14.69 | 0.95 |
| BMI, KG/M2 | 0.05 | 0.07 | 0.79 | 1.05 | 0.92 | 1.20 | 0.43 |
| (Intercept) | 0.36 | 0.51 | 0.71 | 1.43 | 0.53 | 3.86 | 0.48 |
| FSH, mIU/ml | 0.05 | 0.04 | 1.32 | 1.05 | 0.98 | 1.13 | 0.19 |
| (Intercept) | 0.77 | 0.31 | 2.50 | 2.16 | 1.18 | 3.96 | 0.01 |
| Estradiol, pg/ml | 0.00 | 0.01 | 0.87 | 1.00 | 0.99 | 1.02 | 0.39 |
| (Intercept) | 1.08 | 0.38 | 2.87 | 2.95 | 1.41 | 6.19 | 0.00 |
| Progestogen, ng/ml | -0.14 | 0.67 | -0.21 | 0.87 | 0.23 | 3.22 | 0.83 |
| (Intercept) | 1.15 | 0.30 | 3.88 | 3.16 | 1.76 | 5.64 | 0.00 |
| Prolactin, ng/ml | 0.00 | 0.01 | -0.35 | 1.00 | 0.97 | 1.02 | 0.72 |
| (Intercept) | 0.61 | 0.32 | 1.91 | 1.83 | 0.98 | 3.41 | 0.06 |
| LH.base | 0.08 | 0.06 | 1.39 | 1.08 | 0.97 | 1.21 | 0.16 |
| (Intercept) | 1.08 | 0.19 | 5.65 | 2.94 | 2.02 | 4.27 | 0.00 |
| Testosterone, ng/ml | 0.17 | 0.29 | 0.58 | 1.18 | 0.67 | 2.08 | 0.56 |
| (Intercept) | 0.97 | 0.22 | 4.39 | 2.64 | 1.71 | 4.06 | 0.00 |
| AMH.base | 0.03 | 0.09 | 0.34 | 1.03 | 0.86 | 1.23 | 0.74 |
| (Intercept) | 1.17 | 0.34 | 3.39 | 3.21 | 1.63 | 6.31 | 0.00 |
| TSH, mIU/L | -0.06 | 0.11 | -0.52 | 0.94 | 0.76 | 1.18 | 0.60 |
| (Intercept) | 0.86 | 0.21 | 4.05 | 2.35 | 1.56 | 3.56 | 0.00 |
| CA125, U/ML | 0.00 | 0.00 | 1.12 | 1.00 | 1.00 | 1.01 | 0.26 |
| (Intercept) | -0.19 | 1.66 | -0.12 | 0.82 | 0.03 | 21.38 | 0.91 |
| FPG, mmol/L | 0.22 | 0.31 | 0.69 | 1.24 | 0.67 | 2.29 | 0.49 |
| (Intercept) | 0.41 | 0.53 | 0.78 | 1.51 | 0.54 | 4.23 | 0.44 |
| FINS, uIU/ml | 0.08 | 0.05 | 1.53 | 1.09 | 0.98 | 1.21 | 0.13 |
| (Intercept) | 0.68 | 0.26 | 2.62 | 1.98 | 1.19 | 3.29 | 0.01 |
| AFC | 0.05 | 0.03 | 1.65 | 1.05 | 0.99 | 1.11 | 0.10 |
| (Intercept) | 1.59 | 0.33 | 4.81 | 4.91 | 2.57 | 9.39 | 0.00 |
| 25(OH)D≥ 20 ng/ml | -0.79 | 0.38 | -2.08 | 0.45 | 0.22 | 0.95 | 0.04 |
| (Intercept) | -0.42 | 0.24 | -1.74 | 0.66 | 0.41 | 1.05 | 0.08 |
| COH cycle Fresh | 3.02 | 0.42 | 7.18 | 20.40 | 8.95 | 46.48 | 0.00 |
| Cyle.type4 | -14.15 | 882.74 | -0.02 | 0.00 | 0.00 | Inf | 0.99 |
| (Intercept) | 2.08 | 0.40 | 5.19 | 8.00 | 3.65 | 17.55 | 0.00 |
| Protocol type Agonist | 0.32 | 0.72 | 0.44 | 1.37 | 0.33 | 5.68 | 0.66 |
| Protocol type Other | -1.87 | 0.45 | -4.17 | 0.15 | 0.06 | 0.37 | 0.00 |
| **Patient with PCOS** |  |  |  |  |  |  |  |
| (Intercept) | 2.58 | 1.60 | 1.61 | 13.15 | 0.57 | 304.67 | 0.11 |
| Age, years old | -0.05 | 0.05 | -0.85 | 0.96 | 0.86 | 1.06 | 0.39 |
| (Intercept) | 1.61 | 1.10 | 1.47 | 5.00 | 0.58 | 42.80 | 0.14 |
| Education Junior MS | -0.22 | 1.20 | -0.19 | 0.80 | 0.08 | 8.47 | 0.85 |
| Education Senior MS | -1.46 | 1.23 | -1.18 | 0.23 | 0.02 | 2.59 | 0.24 |
| Education Bachelor | -0.14 | 1.14 | -0.13 | 0.87 | 0.09 | 8.12 | 0.90 |
| (Intercept) | 1.39 | 0.29 | 4.80 | 4.00 | 2.27 | 7.04 | 0.00 |
| Infertility type (secondary) | -0.43 | 0.47 | -0.91 | 0.65 | 0.26 | 1.64 | 0.36 |
| (Intercept) | 1.18 | 0.41 | 2.87 | 3.24 | 1.45 | 7.24 | 0.00 |
| Progression, years | 0.01 | 0.09 | 0.14 | 1.01 | 0.85 | 1.21 | 0.89 |
| (Intercept) | 2.45 | 1.52 | 1.61 | 11.53 | 0.59 | 227.04 | 0.11 |
| BMI, KG/M2 | -0.05 | 0.06 | -0.81 | 0.95 | 0.84 | 1.08 | 0.42 |
| (Intercept) | 1.02 | 0.82 | 1.24 | 2.78 | 0.55 | 13.96 | 0.22 |
| FSH, mIU/ml | 0.03 | 0.12 | 0.26 | 1.03 | 0.81 | 1.31 | 0.80 |
| (Intercept) | 0.27 | 0.69 | 0.38 | 1.30 | 0.34 | 5.06 | 0.70 |
| Estradiol, pg/ml | 0.02 | 0.02 | 1.40 | 1.02 | 0.99 | 1.05 | 0.16 |
| (Intercept) | 0.57 | 0.53 | 1.08 | 1.76 | 0.63 | 4.94 | 0.28 |
| Progestogen, ng/ml | 1.26 | 0.98 | 1.29 | 3.54 | 0.52 | 24.10 | 0.20 |
| (Intercept) | 0.89 | 0.42 | 2.11 | 2.44 | 1.06 | 5.59 | 0.04 |
| Prolactin, ng/ml | 0.01 | 0.02 | 0.64 | 1.01 | 0.98 | 1.05 | 0.52 |
| (Intercept) | 1.04 | 0.39 | 2.68 | 2.84 | 1.33 | 6.08 | 0.01 |
| LH.base | 0.02 | 0.04 | 0.56 | 1.02 | 0.95 | 1.11 | 0.58 |
| (Intercept) | 1.40 | 0.57 | 2.44 | 4.05 | 1.32 | 12.50 | 0.01 |
| Testosterone, ng/ml | -0.50 | 1.26 | -0.40 | 0.60 | 0.05 | 7.10 | 0.69 |
| (Intercept) | 1.18 | 0.56 | 2.12 | 3.26 | 1.09 | 9.76 | 0.03 |
| AMH.base | 0.00 | 0.05 | 0.08 | 1.00 | 0.90 | 1.12 | 0.94 |
| (Intercept) | 1.31 | 0.33 | 4.01 | 3.70 | 1.95 | 7.03 | 0.00 |
| TSH, mIU/L | -0.02 | 0.06 | -0.32 | 0.98 | 0.87 | 1.11 | 0.75 |
| (Intercept) | 0.53 | 0.66 | 0.81 | 1.71 | 0.47 | 6.17 | 0.42 |
| CA125, U/ML | 0.06 | 0.04 | 1.44 | 1.06 | 0.98 | 1.16 | 0.15 |
| (Intercept) | 5.01 | 2.63 | 1.90 | 150.00 | 0.86 | 26095.47 | 0.06 |
| FPG, mmol/L | -0.70 | 0.49 | -1.43 | 0.50 | 0.19 | 1.30 | 0.15 |
| (Intercept) | 2.09 | 0.50 | 4.22 | 8.09 | 3.06 | 21.37 | 0.00 |
| FINS, uIU/ml | -0.05 | 0.03 | -1.98 | 0.95 | 0.90 | 1.00 | 0.05 |
| (Intercept) | 1.27 | 0.59 | 2.16 | 3.56 | 1.12 | 11.24 | 0.03 |
| AFC | 0.00 | 0.01 | -0.13 | 1.00 | 0.97 | 1.02 | 0.90 |
| (Intercept) | 1.07 | 0.32 | 3.34 | 2.92 | 1.56 | 5.49 | 0.00 |
| 25(OH)D≥ 20 ng/ml | 0.31 | 0.46 | 0.69 | 1.37 | 0.56 | 3.34 | 0.49 |
| (Intercept) | 0.00 | 1.41 | 0.00 | 1.00 | 0.06 | 15.99 | 1.00 |
| COH cycle Frozen | 0.19 | 1.44 | 0.13 | 1.21 | 0.07 | 20.36 | 0.90 |
| COH cycle Fresh | 19.57 | 1437.07 | 0.01 | 3.14x10^8^ | 0.00 | Inf | 0.99 |
| (Intercept) | 19.57 | 1568.63 | 0.01 | 3.14 x10^6^ | 0.00 | Inf | 0.99 |
| Protocol type Agonist | -17.96 | 1568.63 | -0.01 | 0.00 | 0.00 | Inf | 0.99 |
| Protocol type Other | -19.61 | 1568.63 | -0.01 | 0.00 | 0.00 | Inf | 0.99 |

S.E., standard error; OR, odds ratio; CI, confidence interval; PS, primary school; MS, middle school; BMI, KG/M2, kg/m2, body mass index; PCOS, polycystic ovary syndrome; DOR, diminished ovarian reserve; COH, controlled ovarian hyperstimulation; FSH, follicle-stimulating hormone; LH, luteinizing hormone; TSH, thyroid stimulating hormone; FPG, fasting plasma glucose; FINS, fasting insulin; AFC, antral Follicle Count

# sTable 2. Univariable regression results of biochemical pregnancy

| **Predictor** | **Beta** | **SE** | **Wald** | **OR** | **Lower CI** | **Upper CI** | **P** |
| --- | --- | --- | --- | --- | --- | --- | --- |
| **Entire cohort** |  |  |  |  |  |  |  |
| (Intercept) | 2.14 | 0.48 | 4.45 | 8.48 | 3.31 | 21.73 | 0.00 |
| Age, years old | -0.02 | 0.01 | -1.51 | 0.98 | 0.95 | 1.01 | 0.13 |
| (Intercept) | 1.29 | 0.34 | 3.79 | 3.64 | 1.87 | 7.09 | 0.00 |
| Education Junior MS | 0.34 | 0.37 | 0.92 | 1.40 | 0.68 | 2.86 | 0.36 |
| Education Senior MS | -0.14 | 0.39 | -0.36 | 0.87 | 0.41 | 1.86 | 0.72 |
| Education Bachelor | 0.10 | 0.35 | 0.28 | 1.10 | 0.55 | 2.21 | 0.78 |
| Education MD/PhD | 0.10 | 0.73 | 0.13 | 1.10 | 0.26 | 4.60 | 0.90 |
| (Intercept) | 1.41 | 0.10 | 14.25 | 4.08 | 3.36 | 4.95 | 0.00 |
| Infertility type (secondary) | 0.03 | 0.14 | 0.24 | 1.03 | 0.79 | 1.36 | 0.81 |
| (Intercept) | 1.39 | 0.11 | 12.29 | 4.00 | 3.21 | 4.99 | 0.00 |
| Progression, years | 0.01 | 0.02 | 0.35 | 1.01 | 0.96 | 1.05 | 0.73 |
| (Intercept) | 1.69 | 0.51 | 3.33 | 5.40 | 2.00 | 14.61 | 0.00 |
| BMI, KG/M2 | -0.01 | 0.02 | -0.53 | 0.99 | 0.94 | 1.03 | 0.60 |
| (Intercept) | 1.52 | 0.19 | 7.97 | 4.56 | 3.14 | 6.62 | 0.00 |
| FSH, mIU/ml | -0.01 | 0.02 | -0.30 | 0.99 | 0.95 | 1.04 | 0.77 |
| (Intercept) | 1.37 | 0.13 | 10.49 | 3.93 | 3.05 | 5.08 | 0.00 |
| Estradiol, pg/ml | 0.00 | 0.00 | 0.86 | 1.00 | 1.00 | 1.01 | 0.39 |
| (Intercept) | 1.43 | 0.08 | 18.62 | 4.19 | 3.60 | 4.87 | 0.00 |
| Progestogen, ng/ml | 0.05 | 0.04 | 1.17 | 1.05 | 0.97 | 1.13 | 0.24 |
| (Intercept) | 1.40 | 0.14 | 9.74 | 4.05 | 3.06 | 5.37 | 0.00 |
| Prolactin, ng/ml | 0.00 | 0.01 | 0.64 | 1.00 | 0.99 | 1.02 | 0.52 |
| (Intercept) | 1.29 | 0.13 | 9.97 | 3.63 | 2.81 | 4.67 | 0.00 |
| LH.base | 0.04 | 0.02 | 1.60 | 1.04 | 0.99 | 1.09 | 0.11 |
| (Intercept) | 1.49 | 0.08 | 19.89 | 4.45 | 3.84 | 5.15 | 0.00 |
| Testosterone, ng/ml | 0.00 | 0.02 | -0.18 | 1.00 | 0.96 | 1.03 | 0.86 |
| (Intercept) | 1.16 | 0.12 | 10.08 | 3.20 | 2.55 | 4.01 | 0.00 |
| AMH.base | 0.07 | 0.02 | 2.69 | 1.07 | 1.02 | 1.12 | 0.01 |
| (Intercept) | 1.56 | 0.11 | 13.88 | 4.74 | 3.80 | 5.90 | 0.00 |
| TSH, mIU/L | -0.05 | 0.03 | -1.60 | 0.96 | 0.90 | 1.01 | 0.11 |
| (Intercept) | 1.40 | 0.09 | 14.79 | 4.04 | 3.36 | 4.86 | 0.00 |
| CA125, U/ML | 0.00 | 0.00 | 0.07 | 1.00 | 1.00 | 1.00 | 0.94 |
| (Intercept) | 1.94 | 0.39 | 5.01 | 6.96 | 3.26 | 14.86 | 0.00 |
| FPG, mmol/L | -0.10 | 0.07 | -1.38 | 0.91 | 0.79 | 1.04 | 0.17 |
| (Intercept) | 1.36 | 0.17 | 7.80 | 3.88 | 2.76 | 5.45 | 0.00 |
| FINS, uIU/ml | 0.01 | 0.01 | 0.76 | 1.01 | 0.98 | 1.04 | 0.45 |
| (Intercept) | 1.06 | 0.13 | 8.15 | 2.88 | 2.24 | 3.72 | 0.00 |
| AFC | 0.02 | 0.01 | 3.22 | 1.02 | 1.01 | 1.04 | 0.00 |
| (Intercept) | 1.49 | 0.13 | 11.48 | 4.42 | 3.43 | 5.70 | 0.00 |
| 25(OH)D≥ 20 ng/ml | -0.09 | 0.15 | -0.60 | 0.91 | 0.68 | 1.23 | 0.55 |
| (Intercept) | -1.29 | 0.34 | -3.79 | 0.28 | 0.14 | 0.54 | 0.00 |
| COH cycle Frozen | 2.10 | 0.35 | 5.97 | 8.15 | 4.09 | 16.24 | 0.00 |
| COH cycle Fresh | 4.29 | 0.39 | 11.14 | 73.30 | 34.42 | 156.07 | 0.00 |
| Cyle.type4 | 1.29 | 1.45 | 0.89 | 3.64 | 0.21 | 62.93 | 0.37 |
| (Intercept) | 2.93 | 0.21 | 14.00 | 18.79 | 12.46 | 28.33 | 0.00 |
| Protocol type Agonist | -1.36 | 0.29 | -4.65 | 0.26 | 0.14 | 0.46 | 0.00 |
| Protocol type Other | -2.20 | 0.23 | -9.74 | 0.11 | 0.07 | 0.17 | 0.00 |
| **DOR** |  |  |  |  |  |  |  |
| (Intercept) | 2.88 | 1.20 | 2.41 | 17.81 | 1.71 | 185.92 | 0.02 |
| Age, years old | -0.04 | 0.03 | -1.33 | 0.96 | 0.90 | 1.02 | 0.18 |
| (Intercept) | 2.08 | 1.06 | 1.96 | 8.00 | 1.00 | 63.96 | 0.05 |
| Education Junior MS | -0.84 | 1.09 | -0.77 | 0.43 | 0.05 | 3.67 | 0.44 |
| Education Senior MS | -1.27 | 1.14 | -1.11 | 0.28 | 0.03 | 2.64 | 0.27 |
| Education Bachelor | -0.62 | 1.10 | -0.56 | 0.54 | 0.06 | 4.69 | 0.58 |
| Education MD/PhD | 13.49 | 840.27 | 0.02 | 7.19 x10^5^ | 0.00 | Inf | 0.99 |
| (Intercept) | 1.47 | 0.30 | 4.97 | 4.36 | 2.44 | 7.79 | 0.00 |
| Infertility type (secondary) | -0.23 | 0.36 | -0.64 | 0.79 | 0.39 | 1.61 | 0.52 |
| (Intercept) | 1.32 | 0.26 | 5.00 | 3.75 | 2.23 | 6.29 | 0.00 |
| Progression, years | 0.00 | 0.05 | -0.03 | 1.00 | 0.91 | 1.09 | 0.97 |
| (Intercept) | 0.58 | 1.51 | 0.38 | 1.79 | 0.09 | 34.78 | 0.70 |
| BMI, KG/M2 | 0.04 | 0.07 | 0.49 | 1.04 | 0.90 | 1.19 | 0.62 |
| (Intercept) | 0.70 | 0.55 | 1.27 | 2.01 | 0.68 | 5.91 | 0.20 |
| FSH, mIU/ml | 0.05 | 0.04 | 1.15 | 1.05 | 0.97 | 1.14 | 0.25 |
| (Intercept) | 1.30 | 0.31 | 4.13 | 3.66 | 1.98 | 6.78 | 0.00 |
| Estradiol, pg/ml | 0.00 | 0.01 | 0.05 | 1.00 | 0.99 | 1.01 | 0.96 |
| (Intercept) | 1.52 | 0.41 | 3.72 | 4.58 | 2.06 | 10.21 | 0.00 |
| Progestogen, ng/ml | -0.45 | 0.71 | -0.63 | 0.64 | 0.16 | 2.57 | 0.53 |
| (Intercept) | 1.52 | 0.32 | 4.70 | 4.56 | 2.42 | 8.60 | 0.00 |
| Prolactin, ng/ml | -0.01 | 0.01 | -0.45 | 0.99 | 0.97 | 1.02 | 0.65 |
| (Intercept) | 1.06 | 0.32 | 3.29 | 2.88 | 1.54 | 5.42 | 0.00 |
| LH.base | 0.05 | 0.05 | 0.90 | 1.05 | 0.94 | 1.17 | 0.37 |
| (Intercept) | 1.40 | 0.20 | 6.94 | 4.04 | 2.73 | 6.00 | 0.00 |
| Testosterone, ng/ml | 0.12 | 0.21 | 0.59 | 1.13 | 0.75 | 1.71 | 0.56 |
| (Intercept) | 1.10 | 0.24 | 4.50 | 3.00 | 1.86 | 4.83 | 0.00 |
| AMH.base | 0.14 | 0.12 | 1.20 | 1.15 | 0.92 | 1.44 | 0.23 |
| (Intercept) | 1.56 | 0.37 | 4.19 | 4.76 | 2.30 | 9.89 | 0.00 |
| TSH, mIU/L | -0.09 | 0.12 | -0.78 | 0.91 | 0.72 | 1.15 | 0.43 |
| (Intercept) | 1.23 | 0.23 | 5.42 | 3.43 | 2.20 | 5.36 | 0.00 |
| CA125, U/ML | 0.00 | 0.00 | 0.60 | 1.00 | 0.99 | 1.01 | 0.55 |
| (Intercept) | -0.03 | 1.92 | -0.02 | 0.97 | 0.02 | 41.50 | 0.99 |
| FPG, mmol/L | 0.25 | 0.36 | 0.69 | 1.28 | 0.63 | 2.60 | 0.49 |
| (Intercept) | 1.28 | 0.58 | 2.18 | 3.58 | 1.14 | 11.25 | 0.03 |
| FINS, uIU/ml | 0.03 | 0.06 | 0.51 | 1.03 | 0.92 | 1.15 | 0.61 |
| (Intercept) | 0.73 | 0.29 | 2.52 | 2.08 | 1.18 | 3.69 | 0.01 |
| AFC | 0.09 | 0.04 | 2.24 | 1.09 | 1.01 | 1.18 | 0.02 |
| (Intercept) | 1.96 | 0.38 | 5.20 | 7.12 | 3.40 | 14.93 | 0.00 |
| 25(OH)D≥ 20 ng/ml | -0.87 | 0.43 | -2.06 | 0.42 | 0.18 | 0.96 | 0.04 |
| (Intercept) | 0.03 | 0.23 | 0.12 | 1.03 | 0.65 | 1.63 | 0.91 |
| COH cycle Fresh | 3.00 | 0.48 | 6.26 | 20.11 | 7.86 | 51.43 | 0.00 |
| Cyle.type4 | -15.59 | 1455.40 | -0.01 | 0.00 | 0.00 | Inf | 0.99 |
| (Intercept) | 2.45 | 0.47 | 5.26 | 11.60 | 4.65 | 28.92 | 0.00 |
| Protocol type Agonist | 0.38 | 0.86 | 0.44 | 1.47 | 0.27 | 7.97 | 0.66 |
| Protocol type Other | -1.91 | 0.51 | -3.73 | 0.15 | 0.05 | 0.40 | 0.00 |
| **PCOS** |  |  |  |  |  |  |  |
| (Intercept) | 3.75 | 1.83 | 2.05 | 42.49 | 1.18 | 1529.34 | 0.04 |
| Age, years old | -0.07 | 0.06 | -1.14 | 0.93 | 0.83 | 1.05 | 0.25 |
| (Intercept) | 1.61 | 1.10 | 1.47 | 5.00 | 0.58 | 42.80 | 0.14 |
| Education Junior MS | 0.38 | 1.26 | 0.30 | 1.47 | 0.12 | 17.21 | 0.76 |
| Education Senior MS | -0.41 | 1.28 | -0.32 | 0.67 | 0.05 | 8.16 | 0.75 |
| Education Bachelor | 0.08 | 1.15 | 0.07 | 1.08 | 0.11 | 10.25 | 0.95 |
| (Intercept) | 1.99 | 0.36 | 5.61 | 7.33 | 3.65 | 14.72 | 0.00 |
| Infertility type (secondary) | -0.74 | 0.54 | -1.38 | 0.48 | 0.17 | 1.36 | 0.17 |
| (Intercept) | 1.69 | 0.48 | 3.55 | 5.40 | 2.13 | 13.73 | 0.00 |
| Progression, years | 0.00 | 0.11 | 0.03 | 1.00 | 0.82 | 1.23 | 0.98 |
| (Intercept) | 3.37 | 1.77 | 1.91 | 29.17 | 0.91 | 934.27 | 0.06 |
| BMI, KG/M2 | -0.07 | 0.07 | -0.96 | 0.93 | 0.81 | 1.08 | 0.34 |
| (Intercept) | 1.45 | 0.96 | 1.51 | 4.27 | 0.65 | 28.19 | 0.13 |
| FSH, mIU/ml | 0.04 | 0.14 | 0.27 | 1.04 | 0.78 | 1.38 | 0.79 |
| (Intercept) | 0.82 | 0.80 | 1.03 | 2.27 | 0.48 | 10.76 | 0.30 |
| Estradiol, pg/ml | 0.02 | 0.02 | 1.12 | 1.02 | 0.98 | 1.06 | 0.26 |
| (Intercept) | 0.67 | 0.65 | 1.02 | 1.95 | 0.54 | 7.01 | 0.31 |
| Progestogen, ng/ml | 2.10 | 1.34 | 1.57 | 8.16 | 0.59 | 112.15 | 0.12 |
| (Intercept) | 1.30 | 0.52 | 2.51 | 3.66 | 1.33 | 10.07 | 0.01 |
| Prolactin, ng/ml | 0.02 | 0.02 | 0.67 | 1.02 | 0.97 | 1.06 | 0.50 |
| (Intercept) | 1.27 | 0.46 | 2.77 | 3.58 | 1.45 | 8.83 | 0.01 |
| LH.base | 0.06 | 0.05 | 1.04 | 1.06 | 0.95 | 1.17 | 0.30 |
| (Intercept) | 1.82 | 0.33 | 5.59 | 6.19 | 3.27 | 11.74 | 0.00 |
| Testosterone, ng/ml | -0.32 | 0.41 | -0.77 | 0.73 | 0.32 | 1.63 | 0.44 |
| (Intercept) | 1.51 | 0.64 | 2.34 | 4.51 | 1.28 | 15.96 | 0.02 |
| AMH.base | 0.02 | 0.06 | 0.32 | 1.02 | 0.90 | 1.16 | 0.75 |
| (Intercept) | 1.85 | 0.37 | 4.93 | 6.34 | 3.04 | 13.22 | 0.00 |
| TSH, mIU/L | -0.04 | 0.07 | -0.53 | 0.96 | 0.84 | 1.10 | 0.59 |
| (Intercept) | 1.09 | 0.70 | 1.56 | 2.98 | 0.76 | 11.76 | 0.12 |
| CA125, U/ML | 0.05 | 0.04 | 1.02 | 1.05 | 0.96 | 1.14 | 0.31 |
| (Intercept) | 5.42 | 3.14 | 1.73 | 226.88 | 0.49 | 105978.15 | 0.08 |
| FPG, mmol/L | -0.67 | 0.58 | -1.16 | 0.51 | 0.16 | 1.59 | 0.25 |
| (Intercept) | 2.45 | 0.57 | 4.31 | 11.59 | 3.81 | 35.27 | 0.00 |
| FINS, uIU/ml | -0.05 | 0.03 | -1.52 | 0.95 | 0.90 | 1.01 | 0.13 |
| (Intercept) | 1.14 | 0.68 | 1.68 | 3.13 | 0.83 | 11.89 | 0.09 |
| AFC | 0.01 | 0.02 | 0.83 | 1.01 | 0.98 | 1.05 | 0.41 |
| (Intercept) | 1.41 | 0.35 | 4.00 | 4.10 | 2.05 | 8.18 | 0.00 |
| 25(OH)D≥ 20 ng/ml | 0.61 | 0.53 | 1.15 | 1.85 | 0.65 | 5.27 | 0.25 |
| (Intercept) | 0.00 | 1.41 | 0.00 | 1.00 | 0.06 | 15.99 | 1.00 |
| COH cycle Frozen | 0.84 | 1.45 | 0.58 | 2.31 | 0.14 | 39.31 | 0.56 |
| COH cycle Fresh | 19.57 | 1437.07 | 0.01 | 3.14 x10^8^ | 0.00 | Inf | 0.99 |
| (Intercept) | 19.57 | 1568.63 | 0.01 | 3.14 x10^8^ | 0.00 | Inf | 0.99 |
| Protocol type Agonist | -17.96 | 1568.63 | -0.01 | 0.00 | 0.00 | Inf | 0.99 |
| Protocol type Other | -18.90 | 1568.63 | -0.01 | 0.00 | 0.00 | Inf | 0.99 |

S.E., standard error; OR, odds ratio; CI, confidence interval; PS, primary school; MS, middle school; BMI, KG/M2, kg/m2, body mass index; PCOS, polycystic ovary syndrome; DOR, diminished ovarian reserve; COH, controlled ovarian hyperstimulation; FSH, follicle-stimulating hormone; LH, luteinizing hormone; TSH, thyroid stimulating hormone; FPG, fasting plasma glucose; FINS, fasting insulin; AFC, antral Follicle Count

# sTable 3. Univariable regression results of miscarriage

| **Predictor** | **Beta** | **SE** | **Wald** | **OR** | **Lower CI** | **Upper CI** | **P** |
| --- | --- | --- | --- | --- | --- | --- | --- |
| **Entire cohort** |  |  |  |  |  |  |  |
| (Intercept) | -0.08 | 0.53 | -0.16 | 0.92 | 0.33 | 2.59 | 0.87 |
| Age, years old | 0.05 | 0.02 | 3.24 | 1.06 | 1.02 | 1.09 | 0.00 |
| (Intercept) | 1.84 | 0.41 | 4.52 | 6.29 | 2.83 | 13.96 | 0.00 |
| Education Junior MS | -0.28 | 0.43 | -0.66 | 0.76 | 0.33 | 1.75 | 0.51 |
| Education Senior MS | 0.15 | 0.47 | 0.32 | 1.16 | 0.46 | 2.95 | 0.75 |
| Education Bachelor | -0.28 | 0.42 | -0.67 | 0.76 | 0.33 | 1.72 | 0.50 |
| Education MD/PhD | -0.45 | 0.76 | -0.59 | 0.64 | 0.14 | 2.84 | 0.55 |
| (Intercept) | 1.54 | 0.10 | 14.96 | 4.65 | 3.80 | 5.69 | 0.00 |
| Infertility type (secondary) | 0.19 | 0.15 | 1.29 | 1.21 | 0.91 | 1.62 | 0.20 |
| (Intercept) | 1.48 | 0.12 | 12.27 | 4.41 | 3.48 | 5.59 | 0.00 |
| Progression, years | 0.03 | 0.03 | 1.33 | 1.04 | 0.98 | 1.09 | 0.18 |
| (Intercept) | 1.38 | 0.55 | 2.53 | 3.99 | 1.37 | 11.65 | 0.01 |
| BMI, KG/M2 | 0.01 | 0.02 | 0.46 | 1.01 | 0.96 | 1.06 | 0.65 |
| (Intercept) | 1.39 | 0.21 | 6.51 | 4.03 | 2.65 | 6.13 | 0.00 |
| FSH, mIU/ml | 0.03 | 0.03 | 1.04 | 1.03 | 0.98 | 1.08 | 0.30 |
| (Intercept) | 1.50 | 0.14 | 10.98 | 4.50 | 3.44 | 5.88 | 0.00 |
| Estradiol, pg/ml | 0.00 | 0.00 | 0.86 | 1.00 | 1.00 | 1.01 | 0.39 |
| (Intercept) | 1.58 | 0.08 | 19.89 | 4.85 | 4.15 | 5.66 | 0.00 |
| Progestogen, ng/ml | 0.02 | 0.03 | 0.69 | 1.02 | 0.96 | 1.09 | 0.49 |
| (Intercept) | 1.65 | 0.15 | 11.29 | 5.19 | 3.90 | 6.91 | 0.00 |
| Prolactin, ng/ml | 0.00 | 0.01 | -0.36 | 1.00 | 0.99 | 1.01 | 0.72 |
| (Intercept) | 1.65 | 0.12 | 13.44 | 5.22 | 4.10 | 6.64 | 0.00 |
| LH.base | -0.01 | 0.02 | -0.50 | 0.99 | 0.95 | 1.03 | 0.62 |
| (Intercept) | 1.58 | 0.08 | 20.30 | 4.84 | 4.16 | 5.63 | 0.00 |
| Testosterone, ng/ml | 0.02 | 0.03 | 0.66 | 1.02 | 0.96 | 1.08 | 0.51 |
| (Intercept) | 1.75 | 0.12 | 14.63 | 5.75 | 4.55 | 7.28 | 0.00 |
| AMH.base | -0.03 | 0.02 | -1.34 | 0.97 | 0.93 | 1.01 | 0.18 |
| (Intercept) | 1.73 | 0.12 | 14.42 | 5.64 | 4.46 | 7.14 | 0.00 |
| TSH, mIU/L | -0.03 | 0.03 | -1.11 | 0.97 | 0.91 | 1.03 | 0.27 |
| (Intercept) | 1.54 | 0.11 | 14.30 | 4.64 | 3.76 | 5.73 | 0.00 |
| CA125, U/ML | 0.00 | 0.00 | 1.29 | 1.00 | 1.00 | 1.01 | 0.20 |
| (Intercept) | 0.10 | 0.81 | 0.12 | 1.10 | 0.23 | 5.36 | 0.90 |
| FPG, mmol/L | 0.30 | 0.15 | 1.92 | 1.34 | 0.99 | 1.82 | 0.06 |
| (Intercept) | 1.57 | 0.18 | 8.68 | 4.80 | 3.37 | 6.84 | 0.00 |
| FINS, uIU/ml | 0.00 | 0.01 | 0.32 | 1.00 | 0.98 | 1.03 | 0.75 |
| (Intercept) | 1.80 | 0.13 | 13.50 | 6.04 | 4.65 | 7.84 | 0.00 |
| AFC | -0.01 | 0.01 | -1.64 | 0.99 | 0.98 | 1.00 | 0.10 |
| (Intercept) | 1.63 | 0.14 | 12.00 | 5.09 | 3.90 | 6.64 | 0.00 |
| 25(OH)D≥ 20 ng/ml | 0.01 | 0.16 | 0.04 | 1.01 | 0.73 | 1.38 | 0.97 |
| (Intercept) | 2.46 | 0.52 | 4.73 | 11.75 | 4.23 | 32.61 | 0.00 |
| COH cycle Frozen | -1.62 | 0.53 | -3.06 | 0.20 | 0.07 | 0.56 | 0.00 |
| COH cycle Fresh | 0.51 | 0.55 | 0.92 | 1.66 | 0.56 | 4.89 | 0.36 |
| (Intercept) | 3.02 | 0.22 | 13.86 | 20.59 | 13.42 | 31.59 | 0.00 |
| Protocol type Agonist | -0.62 | 0.35 | -1.75 | 0.54 | 0.27 | 1.08 | 0.08 |
| Protocol type Other | -2.12 | 0.24 | -9.02 | 0.12 | 0.08 | 0.19 | 0.00 |
| **Patient with DOR** |  |  |  |  |  |  |  |
| (Intercept) | -2.77 | 1.63 | -1.70 | 0.06 | 0.00 | 1.53 | 0.09 |
| Age, years old | 0.14 | 0.05 | 2.93 | 1.16 | 1.05 | 1.27 | 0.00 |
| (Intercept) | 1.25 | 0.80 | 1.56 | 3.50 | 0.73 | 16.85 | 0.12 |
| Education Junior MS | 1.09 | 0.89 | 1.22 | 2.98 | 0.52 | 17.19 | 0.22 |
| Education Senior MS | 1.23 | 1.09 | 1.13 | 3.43 | 0.41 | 28.94 | 0.26 |
| Education Bachelor | 0.64 | 0.88 | 0.73 | 1.90 | 0.34 | 10.64 | 0.46 |
| Education MD/PhD | 15.31 | 1385.38 | 0.01 | 4.47 x10^6^ | 0.00 | Inf | 0.99 |
| (Intercept) | 2.13 | 0.37 | 5.68 | 8.37 | 4.02 | 17.43 | 0.00 |
| Infertility type (secondary) | 0.06 | 0.47 | 0.13 | 1.07 | 0.42 | 2.70 | 0.89 |
| (Intercept) | 1.76 | 0.35 | 5.01 | 5.78 | 2.91 | 11.49 | 0.00 |
| Progression, years | 0.11 | 0.08 | 1.36 | 1.11 | 0.95 | 1.30 | 0.18 |
| (Intercept) | 1.25 | 2.07 | 0.60 | 3.47 | 0.06 | 201.27 | 0.55 |
| BMI, KG/M2 | 0.04 | 0.10 | 0.44 | 1.04 | 0.86 | 1.27 | 0.66 |
| (Intercept) | 1.84 | 0.69 | 2.69 | 6.32 | 1.65 | 24.29 | 0.01 |
| FSH, mIU/ml | 0.02 | 0.05 | 0.44 | 1.02 | 0.93 | 1.13 | 0.66 |
| (Intercept) | 2.28 | 0.40 | 5.71 | 9.78 | 4.47 | 21.38 | 0.00 |
| Estradiol, pg/ml | 0.00 | 0.01 | -0.47 | 1.00 | 0.99 | 1.01 | 0.64 |
| (Intercept) | 2.44 | 0.55 | 4.48 | 11.50 | 3.95 | 33.49 | 0.00 |
| Progestogen, ng/ml | -0.63 | 0.92 | -0.68 | 0.53 | 0.09 | 3.24 | 0.50 |
| (Intercept) | 2.22 | 0.43 | 5.17 | 9.20 | 3.97 | 21.32 | 0.00 |
| Prolactin, ng/ml | 0.00 | 0.02 | -0.11 | 1.00 | 0.97 | 1.03 | 0.91 |
| (Intercept) | 2.15 | 0.38 | 5.62 | 8.55 | 4.05 | 18.08 | 0.00 |
| LH.base | 0.00 | 0.06 | -0.05 | 1.00 | 0.89 | 1.11 | 0.96 |
| (Intercept) | 2.21 | 0.26 | 8.56 | 9.15 | 5.51 | 15.19 | 0.00 |
| Testosterone, ng/ml | -0.07 | 0.05 | -1.24 | 0.94 | 0.84 | 1.04 | 0.21 |
| (Intercept) | 2.60 | 0.33 | 7.85 | 13.43 | 7.03 | 25.69 | 0.00 |
| AMH.base | -0.21 | 0.10 | -2.13 | 0.81 | 0.66 | 0.98 | 0.03 |
| (Intercept) | 2.17 | 0.50 | 4.34 | 8.74 | 3.28 | 23.25 | 0.00 |
| TSH, mIU/L | -0.01 | 0.17 | -0.03 | 0.99 | 0.72 | 1.38 | 0.98 |
| (Intercept) | 2.06 | 0.30 | 6.82 | 7.84 | 4.34 | 14.17 | 0.00 |
| CA125, U/ML | 0.00 | 0.01 | 0.41 | 1.00 | 0.99 | 1.01 | 0.68 |
| (Intercept) | -6.17 | 4.00 | -1.54 | 0.00 | 0.00 | 5.34 | 0.12 |
| FPG, mmol/L | 1.64 | 0.78 | 2.10 | 5.14 | 1.11 | 23.70 | 0.04 |
| (Intercept) | 1.51 | 0.72 | 2.09 | 4.55 | 1.10 | 18.81 | 0.04 |
| FINS, uIU/ml | 0.07 | 0.08 | 0.94 | 1.07 | 0.93 | 1.25 | 0.35 |
| (Intercept) | 3.01 | 0.40 | 7.58 | 20.31 | 9.33 | 44.23 | 0.00 |
| AFC | -0.09 | 0.03 | -3.15 | 0.91 | 0.86 | 0.97 | 0.00 |
| (Intercept) | 1.83 | 0.36 | 5.09 | 6.22 | 3.08 | 12.58 | 0.00 |
| 25(OH)D≥ 20 ng/ml | 0.53 | 0.47 | 1.13 | 1.70 | 0.68 | 4.27 | 0.26 |
| (Intercept) | 1.12 | 0.27 | 4.11 | 3.06 | 1.79 | 5.20 | 0.00 |
| COH cycle Fresh | 2.63 | 0.64 | 4.08 | 13.85 | 3.92 | 48.97 | 0.00 |
| Cyle.type4 | 14.45 | 1455.40 | 0.01 | 1.88 x10^6^ | 0.00 | Inf | 0.99 |
| (Intercept) | 3.42 | 0.72 | 4.76 | 30.50 | 7.46 | 124.73 | 0.00 |
| Protocol type Agonist | 0.14 | 1.24 | 0.11 | 1.15 | 0.10 | 13.12 | 0.91 |
| Protocol type Other | -1.93 | 0.76 | -2.52 | 0.15 | 0.03 | 0.65 | 0.01 |
| **Patient with PCOS** |  |  |  |  |  |  |  |
| (Intercept) | -0.62 | 1.98 | -0.31 | 0.54 | 0.01 | 26.19 | 0.76 |
| Age, years old | 0.08 | 0.07 | 1.13 | 1.08 | 0.94 | 1.24 | 0.26 |
| (Intercept) | 1.61 | 1.10 | 1.47 | 5.00 | 0.58 | 42.80 | 0.14 |
| Education Junior MS | 0.05 | 1.22 | 0.04 | 1.05 | 0.10 | 11.56 | 0.97 |
| Education Senior MS | 0.88 | 1.51 | 0.58 | 2.40 | 0.12 | 46.39 | 0.56 |
| Education Bachelor | -0.14 | 1.14 | -0.13 | 0.87 | 0.09 | 8.12 | 0.90 |
| (Intercept) | 1.87 | 0.34 | 5.51 | 6.50 | 3.34 | 12.65 | 0.00 |
| Infertility type (secondary) | -0.62 | 0.53 | -1.18 | 0.54 | 0.19 | 1.51 | 0.24 |
| (Intercept) | 1.29 | 0.47 | 2.76 | 3.65 | 1.46 | 9.13 | 0.01 |
| Progression, years | 0.09 | 0.11 | 0.82 | 1.10 | 0.88 | 1.38 | 0.41 |
| (Intercept) | 1.92 | 1.71 | 1.12 | 6.80 | 0.24 | 195.24 | 0.26 |
| BMI, KG/M2 | -0.01 | 0.07 | -0.16 | 0.99 | 0.86 | 1.14 | 0.87 |
| (Intercept) | 0.57 | 1.02 | 0.56 | 1.77 | 0.24 | 13.08 | 0.58 |
| FSH, mIU/ml | 0.17 | 0.16 | 1.05 | 1.18 | 0.86 | 1.62 | 0.30 |
| (Intercept) | 1.25 | 0.68 | 1.85 | 3.49 | 0.93 | 13.19 | 0.06 |
| Estradiol, pg/ml | 0.01 | 0.01 | 0.59 | 1.01 | 0.98 | 1.04 | 0.56 |
| (Intercept) | 1.59 | 0.28 | 5.59 | 4.91 | 2.81 | 8.58 | 0.00 |
| Progestogen, ng/ml | 0.06 | 0.20 | 0.30 | 1.06 | 0.72 | 1.56 | 0.77 |
| (Intercept) | 1.59 | 0.48 | 3.35 | 4.92 | 1.94 | 12.49 | 0.00 |
| Prolactin, ng/ml | 0.00 | 0.02 | 0.22 | 1.00 | 0.97 | 1.04 | 0.82 |
| (Intercept) | 1.37 | 0.44 | 3.08 | 3.92 | 1.64 | 9.34 | 0.00 |
| LH.base | 0.03 | 0.05 | 0.70 | 1.03 | 0.94 | 1.14 | 0.48 |
| (Intercept) | 1.17 | 0.70 | 1.69 | 3.24 | 0.83 | 12.67 | 0.09 |
| Testosterone, ng/ml | 1.12 | 1.65 | 0.68 | 3.06 | 0.12 | 78.00 | 0.50 |
| (Intercept) | 1.77 | 0.64 | 2.77 | 5.87 | 1.68 | 20.49 | 0.01 |
| AMH.base | -0.01 | 0.06 | -0.24 | 0.99 | 0.87 | 1.11 | 0.81 |
| (Intercept) | 1.87 | 0.37 | 5.12 | 6.50 | 3.17 | 13.29 | 0.00 |
| TSH, mIU/L | -0.06 | 0.06 | -0.94 | 0.94 | 0.83 | 1.07 | 0.35 |
| (Intercept) | 1.45 | 0.44 | 3.27 | 4.26 | 1.79 | 10.13 | 0.00 |
| CA125, U/ML | 0.00 | 0.02 | 0.06 | 1.00 | 0.96 | 1.04 | 0.95 |
| (Intercept) | -0.49 | 2.78 | -0.18 | 0.62 | 0.00 | 141.94 | 0.86 |
| FPG, mmol/L | 0.39 | 0.53 | 0.73 | 1.47 | 0.52 | 4.17 | 0.46 |
| (Intercept) | 1.44 | 0.49 | 2.93 | 4.23 | 1.61 | 11.09 | 0.00 |
| FINS, uIU/ml | 0.01 | 0.03 | 0.23 | 1.01 | 0.95 | 1.07 | 0.82 |
| (Intercept) | 1.03 | 0.68 | 1.51 | 2.80 | 0.73 | 10.66 | 0.13 |
| AFC | 0.02 | 0.02 | 0.99 | 1.02 | 0.98 | 1.05 | 0.32 |
| (Intercept) | 1.68 | 0.39 | 4.37 | 5.37 | 2.53 | 11.43 | 0.00 |
| 25(OH)D≥ 20 ng/ml | -0.07 | 0.52 | -0.14 | 0.93 | 0.34 | 2.57 | 0.89 |
| (Intercept) | 16.57 | 1696.73 | 0.01 | 1.56 x10^7^ | 0.00 | Inf | 0.99 |
| COH cycle Frozen | -15.82 | 1696.73 | -0.01 | 0.00 | 0.00 | Inf | 0.99 |
| COH cycle Fresh | -12.56 | 1696.73 | -0.01 | 0.00 | 0.00 | Inf | 0.99 |
| (Intercept) | 3.83 | 1.01 | 3.79 | 46.00 | 6.34 | 333.56 | 0.00 |
| Protocol type Agonist | 13.74 | 1615.10 | 0.01 | 9.24 x10^5^ | 0.00 | Inf | 0.99 |
| Protocol type Other | -3.26 | 1.06 | -3.09 | 0.04 | 0.00 | 0.30 | 0.00 |

S.E., standard error; OR, odds ratio; CI, confidence interval; PS, primary school; MS, middle school; BMI, KG/M2, kg/m2, body mass index; PCOS, polycystic ovary syndrome; DOR, diminished ovarian reserve; COH, controlled ovarian hyperstimulation; FSH, follicle-stimulating hormone; LH, luteinizing hormone; TSH, thyroid stimulating hormone; FPG, fasting plasma glucose; FINS, fasting insulin; AFC, antral Follicle Count

# sTable 4. Univariable regression results of live birth

| **Predictor** | **Beta** | **SE** | **Wald** | **OR** | **Lower CI** | **Upper CI** | **P** |  |
| --- | --- | --- | --- | --- | --- | --- | --- | --- |
| **Entire cohort** |  |  |  |  |  |  |  |  |
| (Intercept) | 1.13 | 0.40 | 2.79 | 3.09 | 1.40 | 6.83 | 0.01 |  |
| Age, years old | -0.01 | 0.01 | -1.04 | 0.99 | 0.96 | 1.01 | 0.30 |  |
| (Intercept) | 0.97 | 0.31 | 3.10 | 2.64 | 1.43 | 4.89 | 0.00 |  |
| Education Junior MS | -0.10 | 0.33 | -0.30 | 0.91 | 0.47 | 1.74 | 0.77 |  |
| Education Senior MS | -0.67 | 0.35 | -1.89 | 0.51 | 0.26 | 1.03 | 0.06 |  |
| Education Bachelor | -0.28 | 0.32 | -0.85 | 0.76 | 0.40 | 1.43 | 0.39 |  |
| Education MD/PhD | 0.04 | 0.66 | 0.06 | 1.04 | 0.28 | 3.81 | 0.95 |  |
| (Intercept) | 0.75 | 0.08 | 8.89 | 2.11 | 1.79 | 2.49 | 0.00 |  |
| Infertility type (secondary) | -0.07 | 0.12 | -0.59 | 0.93 | 0.74 | 1.17 | 0.55 |  |
| (Intercept) | 0.66 | 0.10 | 6.90 | 1.93 | 1.60 | 2.32 | 0.00 |  |
| Progression, years | 0.01 | 0.02 | 0.68 | 1.01 | 0.98 | 1.05 | 0.50 |  |
| (Intercept) | 1.12 | 0.43 | 2.61 | 3.05 | 1.32 | 7.06 | 0.01 |  |
| BMI, KG/M2 | -0.02 | 0.02 | -0.96 | 0.98 | 0.95 | 1.02 | 0.34 |  |
| (Intercept) | 0.57 | 0.17 | 3.47 | 1.78 | 1.28 | 2.46 | 0.00 |  |
| FSH, mIU/ml | 0.02 | 0.02 | 1.02 | 1.02 | 0.98 | 1.06 | 0.31 |  |
| (Intercept) | 0.69 | 0.11 | 6.58 | 2.00 | 1.63 | 2.46 | 0.00 |  |
| Estradiol, pg/ml | 0.00 | 0.00 | 0.43 | 1.00 | 1.00 | 1.00 | 0.66 |  |
| (Intercept) | 0.72 | 0.06 | 11.42 | 2.06 | 1.82 | 2.33 | 0.00 |  |
| Progestogen, ng/ml | 0.02 | 0.02 | 0.65 | 1.02 | 0.97 | 1.06 | 0.52 |  |
| (Intercept) | 0.60 | 0.12 | 5.02 | 1.83 | 1.44 | 2.31 | 0.00 |  |
| Prolactin, ng/ml | 0.01 | 0.00 | 1.36 | 1.01 | 1.00 | 1.02 | 0.17 |  |
| (Intercept) | 0.61 | 0.10 | 5.95 | 1.85 | 1.51 | 2.26 | 0.00 |  |
| LH.base | 0.03 | 0.02 | 1.38 | 1.03 | 0.99 | 1.06 | 0.17 |  |
| (Intercept) | 0.74 | 0.06 | 11.94 | 2.11 | 1.86 | 2.38 | 0.00 |  |
| Testosterone, ng/ml | 0.01 | 0.02 | 0.58 | 1.01 | 0.97 | 1.05 | 0.56 |  |
| (Intercept) | 0.64 | 0.09 | 6.76 | 1.90 | 1.58 | 2.29 | 0.00 |  |
| AMH.base | 0.02 | 0.02 | 0.89 | 1.02 | 0.98 | 1.05 | 0.37 |  |
| (Intercept) | 0.78 | 0.10 | 8.03 | 2.18 | 1.80 | 2.64 | 0.00 |  |
| TSH, mIU/L | -0.02 | 0.03 | -0.92 | 0.98 | 0.93 | 1.03 | 0.36 |  |
| (Intercept) | 0.70 | 0.08 | 8.76 | 2.01 | 1.72 | 2.35 | 0.00 |  |
| CA125, U/ML | 0.00 | 0.00 | 0.05 | 1.00 | 1.00 | 1.00 | 0.96 |  |
| (Intercept) | 1.31 | 0.37 | 3.52 | 3.71 | 1.79 | 7.70 | 0.00 |  |
| FPG, mmol/L | -0.11 | 0.07 | -1.63 | 0.89 | 0.78 | 1.02 | 0.10 |  |
| (Intercept) | 0.85 | 0.14 | 5.94 | 2.34 | 1.77 | 3.10 | 0.00 |  |
| FINS, uIU/ml | -0.01 | 0.01 | -0.63 | 0.99 | 0.97 | 1.02 | 0.53 |  |
| (Intercept) | 0.64 | 0.11 | 6.03 | 1.90 | 1.54 | 2.34 | 0.00 |  |
| AFC | 0.00 | 0.00 | 0.87 | 1.00 | 0.99 | 1.01 | 0.38 |  |
| (Intercept) | 0.74 | 0.11 | 6.88 | 2.09 | 1.70 | 2.58 | 0.00 |  |
| 25(OH)D≥ 20 ng/ml | -0.04 | 0.13 | -0.31 | 0.96 | 0.75 | 1.24 | 0.76 |  |
| (Intercept) | -1.54 | 0.37 | -4.19 | 0.21 | 0.10 | 0.44 | 0.00 |  |
| COH cycle Frozen | 1.28 | 0.38 | 3.41 | 3.61 | 1.73 | 7.55 | 0.00 |  |
| COH cycle Fresh | 3.95 | 0.39 | 10.04 | 51.75 | 23.96 | 111.78 | 0.00 |  |
| Cyle.type4 | 1.54 | 1.46 | 1.05 | 4.67 | 0.27 | 81.80 | 0.29 |  |
| (Intercept) | 2.39 | 0.17 | 14.44 | 10.87 | 7.87 | 15.03 | 0.00 |  |
| Protocol type Agonist | -1.18 | 0.25 | -4.80 | 0.31 | 0.19 | 0.50 | 0.00 |  |
| Protocol type Other | -2.66 | 0.18 | -14.50 | 0.07 | 0.05 | 0.10 | 0.00 |  |
| **Patients with DOR** |  |  |  |  |  |  |  |  |
| (Intercept) | 1.27 | 1.05 | 1.21 | 3.56 | 0.46 | 27.65 | 0.23 |  |
| Age, years old | -0.01 | 0.03 | -0.38 | 0.99 | 0.93 | 1.05 | 0.70 |  |
| (Intercept) | 2.08 | 1.06 | 1.96 | 8.00 | 1.00 | 63.96 | 0.05 |  |
| Education Junior MS | -1.05 | 1.09 | -0.96 | 0.35 | 0.04 | 2.98 | 0.34 |  |
| Education Senior MS | -1.93 | 1.13 | -1.70 | 0.15 | 0.02 | 1.34 | 0.09 |  |
| Education Bachelor | -1.32 | 1.09 | -1.21 | 0.27 | 0.03 | 2.27 | 0.23 |  |
| Education MD/PhD | -1.39 | 1.62 | -0.86 | 0.25 | 0.01 | 5.98 | 0.39 |  |
| (Intercept) | 0.82 | 0.25 | 3.26 | 2.26 | 1.38 | 3.69 | 0.00 |  |
| Infertility type (secondary) | 0.10 | 0.32 | 0.30 | 1.10 | 0.59 | 2.05 | 0.76 |  |
| (Intercept) | 1.11 | 0.24 | 4.60 | 3.02 | 1.89 | 4.84 | 0.00 |  |
| Progression, years | -0.05 | 0.04 | -1.33 | 0.95 | 0.88 | 1.03 | 0.18 |  |
| (Intercept) | -0.40 | 1.38 | -0.29 | 0.67 | 0.04 | 10.00 | 0.77 |  |
| BMI, KG/M2 | 0.06 | 0.07 | 0.93 | 1.06 | 0.93 | 1.21 | 0.35 |  |
| (Intercept) | 0.17 | 0.49 | 0.36 | 1.19 | 0.46 | 3.11 | 0.72 |  |
| FSH, mIU/ml | 0.05 | 0.04 | 1.44 | 1.05 | 0.98 | 1.13 | 0.15 |  |
| (Intercept) | 0.65 | 0.30 | 2.20 | 1.91 | 1.07 | 3.41 | 0.03 |  |
| Estradiol, pg/ml | 0.00 | 0.01 | 0.81 | 1.00 | 0.99 | 1.01 | 0.42 |  |
| (Intercept) | 1.00 | 0.37 | 2.72 | 2.71 | 1.32 | 5.55 | 0.01 |  |
| Progestogen, ng/ml | -0.27 | 0.64 | -0.42 | 0.76 | 0.22 | 2.70 | 0.68 |  |
| (Intercept) | 0.85 | 0.29 | 2.92 | 2.33 | 1.32 | 4.12 | 0.00 |  |
| Prolactin, ng/ml | 0.00 | 0.01 | 0.22 | 1.00 | 0.98 | 1.03 | 0.82 |  |
| (Intercept) | 0.63 | 0.28 | 2.25 | 1.87 | 1.08 | 3.24 | 0.02 |  |
| LH.base | 0.04 | 0.05 | 0.96 | 1.04 | 0.96 | 1.14 | 0.34 |  |
| (Intercept) | 0.90 | 0.18 | 4.97 | 2.45 | 1.72 | 3.50 | 0.00 |  |
| Testosterone, ng/ml | 0.16 | 0.25 | 0.65 | 1.17 | 0.73 | 1.90 | 0.51 |  |
| (Intercept) | 0.90 | 0.21 | 4.20 | 2.45 | 1.61 | 3.72 | 0.00 |  |
| AMH.base | -0.01 | 0.08 | -0.14 | 0.99 | 0.84 | 1.17 | 0.89 |  |
| (Intercept) | 0.83 | 0.33 | 2.48 | 2.29 | 1.19 | 4.41 | 0.01 |  |
| TSH, mIU/L | 0.01 | 0.11 | 0.11 | 1.01 | 0.81 | 1.26 | 0.92 |  |
| (Intercept) | 0.70 | 0.21 | 3.40 | 2.01 | 1.35 | 3.02 | 0.00 |  |
| CA125, U/ML | 0.01 | 0.00 | 1.35 | 1.01 | 1.00 | 1.01 | 0.18 |  |
| (Intercept) | -0.31 | 1.59 | -0.19 | 0.74 | 0.03 | 16.58 | 0.85 |  |
| FPG, mmol/L | 0.21 | 0.30 | 0.71 | 1.24 | 0.69 | 2.22 | 0.48 |  |
| (Intercept) | 0.59 | 0.50 | 1.19 | 1.81 | 0.68 | 4.80 | 0.23 |  |
| FINS, uIU/ml | 0.04 | 0.05 | 0.79 | 1.04 | 0.95 | 1.14 | 0.43 |  |
| (Intercept) | 0.69 | 0.25 | 2.81 | 1.99 | 1.23 | 3.23 | 0.01 |  |
| AFC | 0.03 | 0.03 | 0.99 | 1.03 | 0.98 | 1.08 | 0.32 |  |
| (Intercept) | 1.20 | 0.29 | 4.09 | 3.33 | 1.87 | 5.94 | 0.00 |  |
| 25(OH)D≥ 20 ng/ml | -0.47 | 0.35 | -1.35 | 0.63 | 0.32 | 1.23 | 0.18 |  |
| (Intercept) | -0.65 | 0.25 | -2.64 | 0.52 | 0.32 | 0.84 | 0.01 |  |
| COH cycle Fresh | 3.03 | 0.40 | 7.58 | 20.77 | 9.48 | 45.51 | 0.00 |  |
| Cyle.type4 | -13.91 | 882.74 | -0.02 | 0.00 | 0.00 | Inf | 0.99 |  |
| (Intercept) | 1.93 | 0.38 | 5.09 | 6.87 | 3.27 | 14.43 | 0.00 |  |
| Protocol type Agonist | 0.15 | 0.65 | 0.23 | 1.16 | 0.32 | 4.17 | 0.82 |  |
| Protocol type Other | -1.89 | 0.43 | -4.40 | 0.15 | 0.07 | 0.35 | 0.00 |  |
| **Patients with PCOS** | |  |  |  |  |  |  |  |
| (Intercept) | 2.70 | 1.52 | 1.77 | 14.95 | 0.75 | 296.25 | 0.08 |  |
| Age, years old | -0.06 | 0.05 | -1.14 | 0.94 | 0.85 | 1.04 | 0.25 |  |
| (Intercept) | 1.61 | 1.10 | 1.47 | 5.00 | 0.58 | 42.80 | 0.14 |  |
| Education Junior MS | -0.46 | 1.19 | -0.38 | 0.63 | 0.06 | 6.54 | 0.70 |  |
| Education Senior MS | -1.76 | 1.23 | -1.44 | 0.17 | 0.02 | 1.91 | 0.15 |  |
| Education Bachelor | -0.43 | 1.13 | -0.38 | 0.65 | 0.07 | 6.04 | 0.71 |  |
| (Intercept) | 1.15 | 0.27 | 4.26 | 3.17 | 1.86 | 5.38 | 0.00 |  |
| Infertility type (secondary) | -0.46 | 0.45 | -1.03 | 0.63 | 0.26 | 1.51 | 0.30 |  |
| (Intercept) | 0.80 | 0.39 | 2.07 | 2.23 | 1.04 | 4.76 | 0.04 |  |
| Progression, years | 0.05 | 0.09 | 0.55 | 1.05 | 0.88 | 1.25 | 0.58 |  |
| (Intercept) | 2.16 | 1.43 | 1.51 | 8.69 | 0.53 | 143.55 | 0.13 |  |
| BMI, KG/M2 | -0.05 | 0.06 | -0.83 | 0.95 | 0.85 | 1.07 | 0.41 |  |
| (Intercept) | 1.35 | 0.75 | 1.79 | 3.84 | 0.88 | 16.76 | 0.07 |  |
| FSH, mIU/ml | -0.06 | 0.11 | -0.51 | 0.95 | 0.76 | 1.17 | 0.61 |  |
| (Intercept) | 0.31 | 0.61 | 0.51 | 1.37 | 0.41 | 4.55 | 0.61 |  |
| Estradiol, pg/ml | 0.01 | 0.01 | 1.12 | 1.02 | 0.99 | 1.04 | 0.26 |  |
| (Intercept) | 0.42 | 0.48 | 0.87 | 1.52 | 0.59 | 3.89 | 0.39 |  |
| Progestogen, ng/ml | 1.06 | 0.87 | 1.23 | 2.90 | 0.53 | 15.86 | 0.22 |  |
| (Intercept) | 0.53 | 0.42 | 1.26 | 1.69 | 0.75 | 3.84 | 0.21 |  |
| Prolactin, ng/ml | 0.02 | 0.02 | 0.94 | 1.02 | 0.98 | 1.05 | 0.35 |  |
| (Intercept) | 1.01 | 0.36 | 2.78 | 2.75 | 1.35 | 5.59 | 0.01 |  |
| LH.base | 0.00 | 0.04 | -0.10 | 1.00 | 0.93 | 1.07 | 0.92 |  |
| (Intercept) | 1.53 | 0.60 | 2.53 | 4.60 | 1.41 | 15.01 | 0.01 |  |
| Testosterone, ng/ml | -1.41 | 1.32 | -1.07 | 0.24 | 0.02 | 3.24 | 0.29 |  |
| (Intercept) | 1.22 | 0.53 | 2.30 | 3.39 | 1.20 | 9.61 | 0.02 |  |
| AMH.base | -0.03 | 0.05 | -0.50 | 0.97 | 0.88 | 1.08 | 0.62 |  |
| (Intercept) | 0.98 | 0.31 | 3.13 | 2.66 | 1.44 | 4.89 | 0.00 |  |
| TSH, mIU/L | 0.00 | 0.06 | 0.07 | 1.00 | 0.89 | 1.14 | 0.94 |  |
| (Intercept) | 0.28 | 0.61 | 0.47 | 1.33 | 0.40 | 4.35 | 0.64 |  |
| CA125, U/ML | 0.06 | 0.04 | 1.55 | 1.06 | 0.98 | 1.15 | 0.12 |  |
| (Intercept) | 4.46 | 2.44 | 1.82 | 86.28 | 0.72 | 10396.08 | 0.07 |  |
| FPG, mmol/L | -0.64 | 0.45 | -1.42 | 0.53 | 0.22 | 1.28 | 0.16 |  |
| (Intercept) | 1.92 | 0.47 | 4.08 | 6.84 | 2.72 | 17.22 | 0.00 |  |
| FINS, uIU/ml | -0.06 | 0.03 | -2.19 | 0.94 | 0.90 | 0.99 | 0.03 |  |
| (Intercept) | 1.35 | 0.56 | 2.42 | 3.87 | 1.29 | 11.55 | 0.02 |  |
| AFC | -0.01 | 0.01 | -0.78 | 0.99 | 0.97 | 1.01 | 0.44 |  |
| (Intercept) | 0.78 | 0.30 | 2.59 | 2.19 | 1.21 | 3.95 | 0.01 |  |
| 25(OH)D≥ 20 ng/ml | 0.41 | 0.43 | 0.95 | 1.50 | 0.65 | 3.48 | 0.34 |  |
| (Intercept) | 0.00 | 1.41 | 0.00 | 1.00 | 0.06 | 15.99 | 1.00 |  |
| COH cycle Frozen | -0.19 | 1.44 | -0.13 | 0.83 | 0.05 | 13.94 | 0.90 |  |
| COH cycle Fresh | 19.57 | 1437.07 | 0.01 | 3.14 x10^8^ | 0.00 | Inf | 0.99 |  |
| (Intercept) | 19.57 | 1568.63 | 0.01 | 3.14 x10^8^ | 0.00 | Inf | 0.99 |  |
| Protocol type Agonist | -17.96 | 1568.63 | -0.01 | 0.00 | 0.00 | Inf | 0.99 |  |
| Protocol type Other | -20.04 | 1568.63 | -0.01 | 0.00 | 0.00 | Inf | 0.99 |  |

S.E., standard error; OR, odds ratio; CI, confidence interval; PS, primary school; MS, middle school; BMI, KG/M2, kg/m2, body mass index; PCOS, polycystic ovary syndrome; DOR, diminished ovarian reserve; COH, controlled ovarian hyperstimulation; FSH, follicle-stimulating hormone; LH, luteinizing hormone; TSH, thyroid stimulating hormone; FPG, fasting plasma glucose; FINS, fasting insulin; AFC, antral Follicle Count

# sTable 5. Multivariable regressions for clinical pregnancy

| **Predictor** | **Beta** | **SE** | **Wald** | **OR** | **Lower CI** | **Upper CI** | **P** |
| --- | --- | --- | --- | --- | --- | --- | --- |
| **Entire cohort** |  |  |  |  |  |  |  |
| (Intercept) | -2.89 | 0.64 | -4.54 | 0.06 | 0.02 | 0.19 | 0.00 |
| 25(OH)D≥ 20 ng/ml | 0.03 | 0.16 | 0.20 | 1.03 | 0.75 | 1.42 | 0.84 |
| AMH.base | 0.10 | 0.03 | 3.89 | 1.10 | 1.05 | 1.16 | 0.00 |
| COH cycle Frozen | 1.52 | 0.45 | 3.37 | 4.57 | 1.89 | 11.06 | 0.00 |
| COH cycle Fresh | 5.04 | 0.58 | 8.69 | 154.82 | 49.67 | 482.59 | 0.00 |
| Cyle.type4 | -11.19 | 535.41 | -0.02 | 0.00 | 0.00 | Inf | 0.98 |
| Protocol type Agonist | 0.40 | 0.39 | 1.02 | 1.50 | 0.69 | 3.25 | 0.31 |
| Protocol type Other | 0.93 | 0.54 | 1.72 | 2.55 | 0.88 | 7.38 | 0.09 |
| **Patients with DOR** |  |  |  |  |  |  |  |
| (Intercept) | -1.54 | 1.22 | -1.26 | 0.21 | 0.02 | 2.35 | 0.21 |
| 25(OH)D≥ 20 ng/ml | -1.02 | 0.47 | -2.19 | 0.36 | 0.14 | 0.90 | 0.03 |
| COH cycle Fresh | 4.38 | 1.14 | 3.84 | 80.12 | 8.53 | 752.27 | 0.00 |
| Cyle.type4 | -14.06 | 1455.40 | -0.01 | 0.00 | 0.00 | Inf | 0.99 |
| Protocol type Agonist | 1.05 | 0.92 | 1.15 | 2.86 | 0.48 | 17.25 | 0.25 |
| Protocol type Other | 1.76 | 1.19 | 1.48 | 5.82 | 0.56 | 60.21 | 0.14 |
| **Patients with PCOS** |  |  |  |  |  |  |  |
| (Intercept) | 1.99 | 0.57 | 3.52 | 7.31 | 2.42 | 22.14 | 0.00 |
| 25(OH)D≥ 20 ng/ml | 0.18 | 0.50 | 0.36 | 1.19 | 0.45 | 3.16 | 0.72 |
| FINS, uIU/ml | -0.05 | 0.03 | -1.98 | 0.95 | 0.90 | 1.00 | 0.05 |

S.E., standard error; OR, odds ratio; CI, confidence interval; PS, primary school; MS, middle school; BMI, KG/M2, kg/m2, body mass index; PCOS, polycystic ovary syndrome; DOR, diminished ovarian reserve; COH, controlled ovarian hyperstimulation; FSH, follicle-stimulating hormone; LH, luteinizing hormone; TSH, thyroid stimulating hormone; FPG, fasting plasma glucose; FINS, fasting insulin; AFC, antral Follicle Count

# sTable 6. Multivariable regressions for biochemical pregnancy

| **Predictor** | **Beta** | **SE** | **Wald** | **OR** | **Lower CI** | **Upper CI** | **P** |
| --- | --- | --- | --- | --- | --- | --- | --- |
| **Entire cohort** |  |  |  |  |  |  |  |
| (Intercept) | -2.99 | 0.67 | -4.43 | 0.05 | 0.01 | 0.19 | 0.00 |
| 25(OH)D≥ 20 ng/ml | 0.05 | 0.18 | 0.27 | 1.05 | 0.74 | 1.49 | 0.78 |
| AMH.base | 0.01 | 0.04 | 0.28 | 1.01 | 0.93 | 1.10 | 0.78 |
| AFC | 0.04 | 0.01 | 3.26 | 1.04 | 1.02 | 1.07 | 0.00 |
| COH cycle Frozen | 2.19 | 0.47 | 4.71 | 8.93 | 3.59 | 22.25 | 0.00 |
| COH cycle Fresh | 5.21 | 0.59 | 8.84 | 183.47 | 57.75 | 582.80 | 0.00 |
| Cyle.type4 | -10.97 | 535.41 | -0.02 | 0.00 | 0.00 | Inf | 0.98 |
| Protocol type Agonist | 0.21 | 0.43 | 0.48 | 1.23 | 0.53 | 2.89 | 0.63 |
| Protocol type Other | 0.73 | 0.57 | 1.28 | 2.07 | 0.68 | 6.28 | 0.20 |
| **Patients with DOR** |  |  |  |  |  |  |  |
| (Intercept) | -3.33 | 1.46 | -2.29 | 0.04 | 0.00 | 0.62 | 0.02 |
| 25(OH)D≥ 20 ng/ml | -0.95 | 0.56 | -1.71 | 0.39 | 0.13 | 1.15 | 0.09 |
| AFC | 0.19 | 0.05 | 3.56 | 1.21 | 1.09 | 1.35 | 0.00 |
| COH cycle Fresh | 5.16 | 1.30 | 3.97 | 174.93 | 13.64 | 2243.50 | 0.00 |
| Cyle.type4 | -14.07 | 1455.40 | -0.01 | 0.00 | 0.00 | Inf | 0.99 |
| Protocol type Agonist | 2.21 | 1.33 | 1.66 | 9.10 | 0.67 | 122.89 | 0.10 |
| Protocol type Other | 2.33 | 1.35 | 1.73 | 10.31 | 0.73 | 144.74 | 0.08 |
| **Patients with PCOS** |  |  |  |  |  |  |  |
| (Intercept) | -1.04 | 0.92 | -1.13 | 0.35 | 0.06 | 2.15 | 0.26 |
| 25(OH)D≥ 20 ng/ml | 0.62 | 0.63 | 0.99 | 1.86 | 0.54 | 6.37 | 0.32 |
| Progestogen, ng/ml | 2.84 | 1.82 | 1.57 | 17.19 | 0.49 | 604.47 | 0.12 |

S.E., standard error; OR, odds ratio; CI, confidence interval; PS, primary school; MS, middle school; BMI, KG/M2, kg/m2, body mass index; PCOS, polycystic ovary syndrome; DOR, diminished ovarian reserve; COH, controlled ovarian hyperstimulation; FSH, follicle-stimulating hormone; LH, luteinizing hormone; TSH, thyroid stimulating hormone; FPG, fasting plasma glucose; FINS, fasting insulin; AFC, antral Follicle Count

# sTable 7. Multivariable regressions for miscarriage

| **Predictor** | **Beta** | **SE** | **Wald** | **OR** | **Lower CI** | **Upper CI** | **P** |
| --- | --- | --- | --- | --- | --- | --- | --- |
| **Entire cohort** |  |  |  |  |  |  |  |
| (Intercept) | 1.18 | 0.87 | 1.35 | 3.26 | 0.59 | 18.09 | 0.18 |
| 25(OH)D≥ 20 ng/ml | 0.02 | 0.17 | 0.12 | 1.02 | 0.73 | 1.44 | 0.90 |
| Age, years old | 0.05 | 0.02 | 2.68 | 1.05 | 1.01 | 1.09 | 0.01 |
| COH cycle Frozen | -1.60 | 0.57 | -2.83 | 0.20 | 0.07 | 0.61 | 0.00 |
| COH cycle Fresh | 0.31 | 0.63 | 0.49 | 1.37 | 0.40 | 4.72 | 0.62 |
| Cyle.type4 | 10.77 | 535.41 | 0.02 | 47731.61 | 0.00 | Inf | 0.98 |
| Protocol type Agonist | -0.38 | 0.39 | -0.97 | 0.68 | 0.32 | 1.47 | 0.33 |
| Protocol type Other | -0.33 | 0.53 | -0.62 | 0.72 | 0.25 | 2.05 | 0.54 |
| **Patients with DOR** |  |  |  |  |  |  |  |
| (Intercept) | 3.41 | 2317.33 | 0.00 | 30.33 | 0.00 | Inf | 1.00 |
| 25(OH)D≥ 20 ng/ml | 0.86 | 0.75 | 1.16 | 2.37 | 0.55 | 10.22 | 0.25 |
| Age, years old | 0.12 | 0.08 | 1.53 | 1.13 | 0.97 | 1.31 | 0.13 |
| AMH.base | 0.30 | 0.29 | 1.01 | 1.35 | 0.76 | 2.39 | 0.31 |
| FPG, mmol/L | 2.07 | 1.18 | 1.75 | 7.96 | 0.78 | 80.80 | 0.08 |
| AFC | -0.14 | 0.09 | -1.57 | 0.87 | 0.73 | 1.03 | 0.12 |
| COH cycle Fresh | 2.63 | 1.44 | 1.83 | 13.83 | 0.83 | 231.36 | 0.07 |
| Cyle.type4 | 16.34 | 17730.37 | 0.00 | 1.25x10^7^ | 0.00 | Inf | 1.00 |
| Protocol type Agonist | -16.61 | 2317.32 | -0.01 | 0.00 | 0.00 | Inf | 0.99 |
| Protocol type Other | -16.94 | 2317.32 | -0.01 | 0.00 | 0.00 | Inf | 0.99 |
| **Patients with PCOS** |  |  |  |  |  |  |  |
| (Intercept) | 3.91 | 1.06 | 3.69 | 49.85 | 6.24 | 398.15 | 0.00 |
| 25(OH)D≥ 20 ng/ml | -0.15 | 0.58 | -0.26 | 0.86 | 0.27 | 2.70 | 0.79 |
| Protocol type Agonist | 13.76 | 1614.40 | 0.01 | 9.46x10^5^ | 0.00 | Inf | 0.99 |
| Protocol type Other | -3.26 | 1.06 | -3.09 | 0.04 | 0.00 | 0.30 | 0.00 |

S.E., standard error; OR, odds ratio; CI, confidence interval; PS, primary school; MS, middle school; BMI, KG/M2, kg/m2, body mass index; PCOS, polycystic ovary syndrome; DOR, diminished ovarian reserve; COH, controlled ovarian hyperstimulation; FSH, follicle-stimulating hormone; LH, luteinizing hormone; TSH, thyroid stimulating hormone; FPG, fasting plasma glucose; FINS, fasting insulin; AFC, antral Follicle Count

# sTable 8. Multivariable regressions for live birth

| **Predictor** | **Beta** | **SE** | **Wald** | **OR** | **Lower CI** | **Upper CI** | **P** |
| --- | --- | --- | --- | --- | --- | --- | --- |
| **Entire cohort** |  |  |  |  |  |  |  |
| (Intercept) | -2.04 | 0.59 | -3.48 | 0.13 | 0.04 | 0.41 | 0.00 |
| 25(OH)D≥ 20 ng/ml | 0.05 | 0.16 | 0.33 | 1.05 | 0.77 | 1.43 | 0.75 |
| COH cycle Frozen | 1.42 | 0.47 | 3.02 | 4.14 | 1.65 | 10.43 | 0.00 |
| COH cycle Fresh | 4.39 | 0.55 | 8.00 | 80.51 | 27.46 | 236.04 | 0.00 |
| Cyle.type4 | -11.43 | 535.41 | -0.02 | 0.00 | 0.00 | Inf | 0.98 |
| Protocol type Agonist | -0.15 | 0.32 | -0.47 | 0.86 | 0.46 | 1.61 | 0.64 |
| Protocol type Other | 0.22 | 0.47 | 0.46 | 1.24 | 0.49 | 3.13 | 0.65 |
| **Patients with DOR** |  |  |  |  |  |  |  |
| (Intercept) | -1.98 | 1.15 | -1.72 | 0.14 | 0.01 | 1.32 | 0.09 |
| 25(OH)D≥ 20 ng/ml | -0.57 | 0.44 | -1.30 | 0.56 | 0.24 | 1.34 | 0.19 |
| COH cycle Fresh | 4.32 | 1.07 | 4.03 | 75.19 | 9.21 | 613.88 | 0.00 |
| Cyle.type4 | -13.72 | 1455.40 | -0.01 | 0.00 | 0.00 | Inf | 0.99 |
| Protocol type Agonist | 0.71 | 0.77 | 0.92 | 2.04 | 0.45 | 9.29 | 0.36 |
| Protocol type Other | 1.65 | 1.11 | 1.48 | 5.22 | 0.59 | 46.44 | 0.14 |
| **Patients with PCOS** |  |  |  |  |  |  |  |
| (Intercept) | 1.79 | 0.53 | 3.36 | 6.01 | 2.11 | 17.12 | 0.00 |
| 25(OH)D≥ 20 ng/ml | 0.23 | 0.47 | 0.49 | 1.26 | 0.50 | 3.17 | 0.62 |
| FINS, uIU/ml | -0.06 | 0.03 | -2.19 | 0.94 | 0.90 | 0.99 | 0.03 |

S.E., standard error; OR, odds ratio; CI, confidence interval; PS, primary school; MS, middle school; BMI, KG/M2, kg/m2, body mass index; PCOS, polycystic ovary syndrome; DOR, diminished ovarian reserve; COH, controlled ovarian hyperstimulation; FSH, follicle-stimulating hormone; LH, luteinizing hormone; TSH, thyroid stimulating hormone; FPG, fasting plasma glucose; FINS, fasting insulin; AFC, antral Follicle Count
